# Supplementary figures and images for: Evaluation of the influenza-like illness sentinel surveillance system: A national perspective in Tanzania from January to December 2019
Source: PLoS One. 2023 Mar 20;18(3):e0283043. doi: 10.1371/journal.pone.0283043 (PMC10027206; doi:10.1371/journal.pone.0283043)

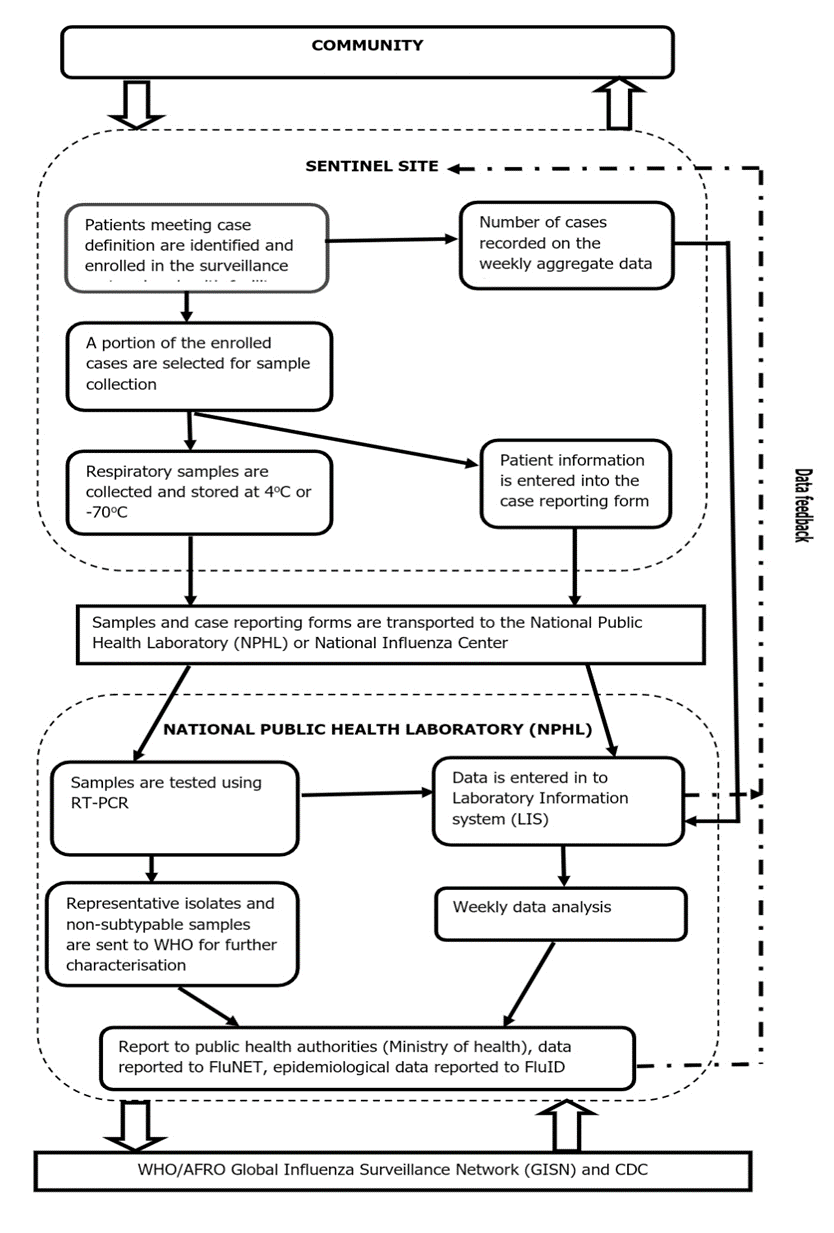

Supplement: S1 Fig — (TIF) [file pone.0283043.s001.tif]
