# Supplementary material for: Evaluation of the influenza-like illness sentinel surveillance system: A national perspective in Tanzania from January to December 2019
Source: PLoS One. 2023 Mar 20;18(3):e0283043. doi: 10.1371/journal.pone.0283043 (PMC10027206; doi:10.1371/journal.pone.0283043)
Supplement: S1 File — (PDF) [file pone.0283043.s002.pdf]

DATA COLLECTION TOOL FOR EVALUATION OF THE NATIONAL INFLUENZA SENTINEL SURVEILLANCE SYSTEM IN TANZANIA, 2021

PART A:

DATA COLLECTION TOOL FOR SURVEILLANCE STAFF AT SENTINEL SITE

Questionnaire number: ……………………………………………

Date: ……………………………………………

Region: ……………………………………………

Health facility: ……………………………………………

Designation of the interviewee: ……………………………………………

Please give your answer for each of the following questions. Read all the answers first and choose the appropriate answer box by circling only one number of each question, and in your opinion respond to the open-ended questions accordingly.

1. How do you identify a person who present with influenza like illness?

1	By use of standard case definition	
2	Observing signs and symptoms	

Others; Mention………………………………………………………………………………………

……………………………………………………………………………………………………

……………………………………………………………………………………………………

2. How do you identify a person who present with severe acute respiratory illness?

1	By use of standard case definition	
2	Observing signs and symptoms	

Others; Mention………………………………………………………………………………………

……………………………………………………………………………………………………

3. Describe the case definition used at your facility……………………………………………………..

……………………………………………………………………………………………………...

4. Is it easy to identify patient by using case definition?

1	YES	
2	NO	

If the answer is NO, where do you think modifications can be made? ……………………………….

…………………………………………………………………………………………………….

…………………………………………………………………………………………………….

5. After you have identified people who meet the standard case definition do you fill a surveillance data collection form?

1	YES	
2	NO	

6. How is the surveillance data collection form?

1	Easy to fill	
2	Complex	

If complex, which part of the form is complex or not clear………………………………………..

…………………………………………………………………………………………………….

……………………………………………………………………………………………………..

7. What are the number of patients admitted for severe acute respiratory illness in 2019?

Month	Number of admitted SARI cases
January	
February	
March	
April	
May	
June	
July	
August	
September	
October	
November	
December	

DATA COLLECTION TOOL FOR EVALUATION OF THE NATIONAL INFLUENZA SENTINEL SURVEILLANCE SYSTEM IN TANZANIA, 2021

8. What is the number of samples collected from patients admitted for severe acute respiratory illness in 2019?

Month	Number of samples collected from SARI cases
January	
February	
March	
April	
May	
June	
July	
August	
September	
October	
November	
December	

9. Which type/s of samples does the surveillance system collect?

1	Nasopharyngeal	
2	Oral pharyngeal	
3	Both (1 & 2)	
4	Throat Swab	

10. Which media do you use after collection of samples?

1	Viral transport media	
2	Cary Blair	
3	Universal transport media	
4	I don't know	

11. After collection of the sample, how do you store?

1	At 2-8 ^{o}C	
2	Under -70 ^{o}C	
3	At room temperature	
4	At -20 ^{o}C	

Others; Specify……………………………………………………………………………………...

…………………………………………………………………………………………………….

12. How long does it take you to transfer specimen to the NIC or NPHL?

1	Twice a week	
2	Once a week	
3	Once a Month	
4	Twice a month	

13. How many samples sent from your facility to NIC or NPHL in 2019?

Month	Number of samples sent to NIC/NPHL
January	
February	
March	
April	
May	
June	
July	
August	
September	
October	
November	
December	

14. How are samples transferred?

1	At 2-8ºC	
2	At -20 ºC	
3	Under -70 ºC	
4	At room temperature	

Others; Specify……………………………………………………………………………………….

………………………………………………………………………………………………………

15. Have you been trained on samples collection and transportation?

1	YES	
2	NO	

If YES, when was your last training? …………………………………………………………...

…….

16. How long does it take to receive the results from NIC or NPHL?

1	One day	
2	One week	
3	Two weeks	
4	One Month	

Others; Specify…………………………………………………………………………………...

……

DATA COLLECTION TOOL FOR EVALUATION OF THE NATIONAL INFLUENZA SENTINEL SURVEILLANCE SYSTEM IN TANZANIA, 2021

PART B

DATA COLLECTION TOOL FOR LABORATORY STAFF AT NATIONAL INFLUENZA CENTRE/NPHL

Questionnaire number: …………………………………………….

Date: …………………………………………….

Region: …………………………………………….

Health facility: …………………………………………….

Designation of the interviewee: …………………………………………….

Please give your answer for each of the following questions. Read all the answers first and choose the appropriate answer box by circling only one number of each question, and in your opinion respond to the open-ended questions accordingly.

1. Is the system able to integrate other surveillance system?

1	YES	
2	NO	

2. Is there any modification needed for the system?

1	YES	
2	NO	

If YES, what modifications can be done?...

……………………………………………………………………………………………………..

……………………………………………………………………………………………………..

3. Does this system meet its objectives?

1	YES	
2	NO	

DATA COLLECTION TOOL FOR EVALUATION OF THE NATIONAL INFLUENZA SENTINEL SURVEILLANCE SYSTEM IN TANZANIA, 2021

4. How do you send feedback /information to the sites?

1	Telephone	
2	Email	
3	EMS	
4	Fax	

5. How is labeling of the specimen and request forms?

1	Always complete	
2	Sometimes not complete	
3	Always not completed	

6. Is there any patient ever diagnosed with highly pathogenic avian influenza from the suspects?

1	YES	
2	NO	

7. Is there any viral variant or outbreak detected by the system?

1	YES	
2	NO	

If YES, Mention it…………………………………………………………………………………

………………………………………………………………………………………………...

8. By which criteria was the diagnosis made?

1	Clinical	
2	Epi-link	
3	Laboratory confirmed	

9. Does the system detect the false positive and false negatives influenza cases?

1	YES	
2	NO	

If YES give the number and possible causes of false positives and false negatives ……………

…………………………………………………………………………………………………

10. Does the system provide estimates of magnitude of morbidity and mortality related influenza?

1	YES	
2	NO	
3	I don’t know	

11. What is the population monitored?

1	YES	
2	NO	
3	I don’t know	

12. Is workforce at the laboratory sufficient?

1	YES	
2	NO	

13. Is training to laboratory staff done?

1	YES	
2	NO	

14. How is the information stored?

1	On papers	
2	Electronic	
3	Both papers and electronic	

15. How are the data being analyzed?

1	Manual	
2	Computer	
3	Both manual and computer	

16. Is the internal quality control and external quality assessment performed in the laboratory?

1	YES	
2	NO	

If YES, How often ……………………………………………………………………………...

…………………………………………………………………………………………………

17. How is the general performance of EQA?

1	≥ 90%	
2	<90%	

18. How many samples received from sentinel sites in 2019?

**Month**	**Number of samples received from sentinel sites**		
	**Sentinel site-1**	**Sentinel site-2**	**Sentinel site-3**
January			
February			
March			
April			
May			
June			
July			
August			
September			
October			
November			
December			

19. How long does it take from receipt of samples to results dispatch?

1	≤ 1 week	
2	>1 week	

If >1 week; what are possible reasons?……...……………………………………………………...

DATA COLLECTION TOOL FOR EVALUATION OF THE NATIONAL INFLUENZA SENTINEL SURVEILLANCE SYSTEM IN TANZANIA, 2021

PART C

DATA COLLECTION TOOL FOR MOHCDGEC OFFICIALS -EPIDEMIOLOGY SECTION

Questionnaire number: ……………………………………………

Date: ……………………………………………

Region: …………………………………………….

Designation of the interviewee: ……………………………………………

Please give your answer for each of the following questions. Read all the answers first and choose the appropriate answer box by circling only one number of each question, and in your opinion respond to the open-ended questions accordingly.

1. Should influenza be under surveillance system?

1	YES	
2	NO	

If YES; why?..

…………………………………………………………………………………………………….

2. Does the system provide estimates of magnitude of morbidity and mortality related influenza?

1	YES	
2	NO	

If YES, explain……………………………………………………………………………………

……………………………………………………………………………………………………..

DATA COLLECTION TOOL FOR EVALUATION OF THE NATIONAL INFLUENZA SENTINEL SURVEILLANCE SYSTEM IN TANZANIA, 2021

3. Is there any modification for the system needed?

1	YES	
2	NO	

If YES, Why?...

……

4. How enough is the information collected by the system?

1	Too much	
2	Too little	
3	Sufficient	

5. Does this system meet its objective?

1	YES	
2	NO	

i. If YES; Explain………………………………………………………………………………...

…………………………………………………………………………………………………

ii. If NO; Explain …………………………………………………………………………………

…………………………………………………………………………………………………

…………………………………………………………………………………………………

6. How are the data generated by the system used?...

…………………………………………………………………………………………………

7. Can the system operate without donor support?

1	YES	
2	NO	

DATA COLLECTION TOOL FOR EVALUATION OF THE NATIONAL INFLUENZA SENTINEL SURVEILLANCE SYSTEM IN TANZANIA, 2021

PART D:
DATA COLLECTION TOOL FOR COLLABORATIVE PARTNERS (CDC/WHO)

Questionnaire number: ……………………………………………

Date: ……………………………………………

Region: ……………………………………………

Designation of the interviewee: ……………………………………………

Please give your answer for each of the following questions. Read all the answers first and choose the appropriate answer box by circling only one number of each question, and in your opinion respond to the open-ended questions accordingly.

1. Is there any modification for the system needed?

1	YES	
2	NO	

If YES, Why?……………………………………………………………………………………………………

……………………………………………………………………………………………………

……………………………………………………………………………………………………

2. How enough is the information collected by the system?

1	Too much	
2	Too little	
3	Sufficient	

DATA COLLECTION TOOL FOR EVALUATION OF THE NATIONAL INFLUENZA SENTINEL SURVEILLANCE SYSTEM IN TANZANIA, 2021

3. How are the data generated by the system used?...

………………………………………………………………………………………………

………………………………………………………………………………………………...

4. Should influenza be under surveillance system?

1	YES	
2	NO	

If YES; why?...

………………………………………………………………………………………………

………………………………………………………………………………………………

5. Does the system provide estimates of magnitude of morbidity and mortality related influenza?

1	YES	
2	NO	

If YES, explain……………………………………………………………………………

………………………………………………………………………………………………..

………………………………………………………………………………………………
