# Supplementary material for: Evaluation of the influenza-like illness sentinel surveillance system: A national perspective in Tanzania from January to December 2019
Source: PLoS One. 2023 Mar 20;18(3):e0283043. doi: 10.1371/journal.pone.0283043 (PMC10027206; doi:10.1371/journal.pone.0283043)
Supplement: S2 File — (PDF) [file pone.0283043.s003.pdf]

CDC
CENTERS FOR DISEASE CONTROL AND PREVENTION

July 27, 2001 / Vol. 50 / No. RR-13

MMWR™
MORBIDITY AND MORTALITY WEEKLY REPORT

Recommendations and Reports

Inside: Continuing Education Examination

Updated Guidelines for Evaluating Public Health Surveillance Systems

Recommendations from the Guidelines Working Group

U.S. DEPARTMENT OF HEALTH AND HUMAN SERVICES
Centers for Disease Control and Prevention (CDC)
Atlanta, GA 30333

The *MMWR* series of publications is published by the Epidemiology Program Office, Centers for Disease Control and Prevention (CDC), U.S. Department of Health and Human Services, Atlanta, GA 30333.

SUGGESTED CITATION

Centers for Disease Control and Prevention. Updated guidelines for evaluating public health surveillance systems: recommendations from the guidelines working group. *MMWR* 2001;50(No. RR-13):[inclusive page numbers].

Centers for Disease Control and Prevention .... Jeffrey P. Koplan, M.D., M.P.H.
Director

The material in this report was prepared for publication by
Epidemiology Program Office .... Stephen B. Thacker, M.D., M.Sc.
Director

Division of Public Health Surveillance and Informatics .... Daniel M. Sosin, M.D., M.P.H.
Director

National Center for HIV, STD, and TB Prevention .... Helene D. Gayle, M.D., M.P.H.
Director

Division of HIV/AIDS Prevention — Surveillance and Epidemiology .... Robert S. Janssen, M.D.
Director

National Center for Injury Prevention and Control .... Suzanne Binder, M.D.
Director

National Center for Chronic Disease Prevention and Health Promotion .... James S. Marks, M.D., M.P.H.
Director

Division of Adult and Community Health .... Gary C. Hogelin, M.P.A.
Director

National Center for Environmental Health .... Richard J. Jackson, M.D., M.P.H.
Director

Division of Environmental Hazards and Health Effects .... Michael A. McGeehin
Director

This report was produced as an *MMWR* serial publication in
Epidemiology Program Office .... Stephen B. Thacker, M.D., M.Sc.
Director

Office of Scientific and Health Communications .... John W. Ward, M.D.
Director
Editor, MMWR *Series*

Recommendations and Reports .... Suzanne M. Hewitt, M.P.A.
Managing Editor

Patricia A. McGee
Project Editor

Morie M. Higgins
Visual Information Specialist

Michele D. Renshaw and Erica R. Shaver
Information Technology Specialists

Contents

Introduction 1
Background 2
Organization of This Report 3
Task A. Engage the Stakeholders in the Evaluation 4
Task B. Describe the Surveillance System to be Evaluated 4
Task C. Focus the Evaluation Design 11
Task D. Gather Credible Evidence Regarding the Performance of the Surveillance System 13
Task E. Justify and State Conclusions, and Make Recommendations 24
Task F. Ensure Use of Evaluation Findings and Share Lessons Learned 25
Summary 25
References 25
Appendices 31
Continuing Education Examination CE-1

Guidelines Working Group

CHAIRMAN

Robert R. German, M.P.H.
Epidemiology Program Office, CDC

ADMINISTRATIVE SUPPORT

Dwight Westmoreland, M.P.A.
Epidemiology Program Office, CDC

MEMBERS

Greg Armstrong, M.D.
National Center for Infectious Diseases CDC

Guthrie S. Birkhead, M.D., M.P.H.
Council of State and Territorial Epidemiologists
New York State Department of Health
Albany, New York

John M. Horan, M.D., M.P.H.
National Center for Injury Prevention and Control, CDC

Guillermo Herrera
National Immunization Program, CDC

Lisa M. Lee, Ph.D.
National Center for HIV, STD and TB Prevention, CDC

Robert L. Milstein, M.P.H.
National Center for Chronic Disease Prevention and Health Promotion, CDC

Carol A. Pertowski, M.D.
National Center for Environmental Health CDC

Michael N. Waller
National Center for Chronic Disease Prevention and Health Promotion, CDC

The following CDC staff members prepared this report:

Robert R. German, M.P.H.
Division of Public Health Surveillance and Informatics
Epidemiology Program Office

Lisa M. Lee, Ph.D.
Division of HIV/AIDS Prevention — Surveillance and Epidemiology
National Center for HIV, STD, and TB Prevention

John M. Horan, M.D., M.P.H.
Office of the Director
National Center for Injury Prevention and Control

Robert L. Milstein, M.P.H.
Office of the Director
National Center for Chronic Disease Prevention and Health Promotion

Carol A. Pertowski, M.D.
Division of Environmental Hazards and Health Effects
National Center for Environmental Health

Michael N. Waller
Division of Adult and Community Health
National Center for Chronic Disease Prevention and Health Promotion

in collaboration with

Guthrie S. Birkhead, M.D., M.P.H.
Council of State and Territorial Epidemiologists
New York State Department of Health
Albany, New York

Additional CDC Contributors

Office of the Director: Karen E. Harris, M.P.H.; Joseph A. Reid, Ph.D; Gladys H. Reynolds, Ph.D., M.S.; Dixie E. Snider, Jr., M.D., M.P.H.
Agency for Toxic Substances and Disease Registry: Wendy E. Kaye, Ph.D.; Robert Spengler, Sc.D.
Epidemiology Program Office: Vilma G. Carande-Kulis, Ph.D., M.S.; Andrew G. Dean, M.D., M.P.H.; Samuel L. Groseclose, D.V.M., M.P.H.; Robert A. Hahn, Ph.D., M.P.H.; Lori Hutwagner, M.S.; Denise Koo, M.D., M.P.H.; R. Gibson Parrish, M.D., M.P.H.; Catherine Schenck-Yglesias, M.H.S.; Daniel M. Sosin, M.D., M.P.H.; Donna F. Stroup, Ph.D., M.Sc.; Stephen B. Thacker, M.D., M.Sc.; G. David Williamson, Ph.D.
National Center for Birth Defects and Developmental Disabilities: Joseph Mulnaire, M.D., M.S.P.H.
National Center for Chronic Disease Prevention and Health Promotion: Terry F. Pechacek, Ph.D; Nancy Stroup, Ph.D.
National Center for Environmental Health: Thomas H. Sinks, Ph.D.
National Center for Health Statistics: Jennifer H. Madans, Ph.D.
National Center for HIV, STD, and TB Prevention: James W. Buehler, M.D.; Meade Morgan, Ph.D.
National Center for Infectious Diseases: Janet K. Nicholson, Ph.D; Jose G. Rigau-Perez, M.D., M.P.H.
National Center for Injury Prevention and Control: Richard L. Ehrenberg, M.D.
National Immunization Program: H. Gay Allen, M.S.P.H.; Roger H. Bernier, Ph.D; Nancy Koughan, D.O., M.P.H., M.H.A.; Sandra W. Roush, M.T., M.P.H.
National Institute for Occupational Safety and Health: Rosemary Sokas, M.D., M.O.H.
Public Health Practice Program Office: William A. Yasnoff, M.D., Ph.D.

Consultants and Contributors

Scientific Workgroup on Health-Related Quality of Life Surveillance
St. Louis University, St. Louis, Missouri

Paul Etkind, Dr.P.H., Massachusetts Department of Public Health, Jamaica Plain, Massachusetts; Annie Fine, M.D., New York City Department of Health, New York City, New York; Julie A. Fletcher, D.V.M, M.P.H. candidate, Emory University, Atlanta, Georgia; Daniel J. Friedman, Ph.D., Massachusetts Department of Public Health, Boston, Massachusetts; Richard S. Hopkins, M.D., M.S.P.H., Florida Department of Health, Tallahassee, Florida; Steven C. MacDonald, Ph.D., M.P.H., Washington State Department of Health, Olympia, Washington; Elroy D. Mann, D.V.M., M.Sc., Health Canada, Ottawa, Canada; S. Potjaman, M.D., Government of Thailand, Bangkok, Thailand; Marcel E. Salive, M.D., M.P.H., National Institutes of Health, Bethesda, Maryland.

Updated Guidelines for Evaluating Public Health Surveillance Systems

Recommendations from the Guidelines Working Group

Summary

The purpose of evaluating public health surveillance systems is to ensure that problems of public health importance are being monitored efficiently and effectively. CDC's Guidelines for Evaluating Surveillance Systems *are being updated to address the need for a) the integration of surveillance and health information systems, b) the establishment of data standards, c) the electronic exchange of health data, and d) changes in the objectives of public health surveillance to facilitate the response of public health to emerging health threats (e.g., new diseases). This report provides updated guidelines for evaluating surveillance systems based on CDC's* Framework for Program Evaluation in Public Health*, research and discussion of concerns related to public health surveillance systems, and comments received from the public health community. The guidelines in this report describe many tasks and related activities that can be applied to public health surveillance systems.*

INTRODUCTION

In 1988, CDC published *Guidelines for Evaluating Surveillance Systems* (*1*) to promote the best use of public health resources through the development of efficient and effective public health surveillance systems. CDC's *Guidelines for Evaluating Surveillance Systems* are being updated to address the need for a) the integration of surveillance and health information systems, b) the establishment of data standards, c) the electronic exchange of health data, and d) changes in the objectives of public health surveillance to facilitate the response of public health to emerging health threats (e.g., new diseases). For example, CDC, with the collaboration of state and local health departments, is implementing the National Electronic Disease Surveillance System (NEDSS) to better manage and enhance the large number of current surveillance systems and allow the public health community to respond more quickly to public health threats (e.g., outbreaks of emerging infectious diseases and bioterrorism) (*2*). When NEDSS is completed, it will electronically integrate and link together several types of surveillance systems with the use of standard data formats; a communications infrastructure built on principles of public health informatics; and agreements on data access, sharing, and confidentiality. In addition, the Health Insurance Portability and Accountability Act of 1996 (HIPAA) mandates that the United States adopt national uniform standards for electronic transactions related to health insurance enrollment and eligibility, health-care encounters, and health insurance claims; for identifiers for health-care providers, payers and individuals, as well as code sets and classification systems used in these transactions; and for security of these transactions (*3*). The electronic exchange of health data inherently involves the protection of patient privacy.

Based on CDC's *Framework for Program Evaluation in Public Health* (*4*), research and discussion of concerns related to public health surveillance systems, and comments received from the public health community, this report provides updated guidelines for evaluating public health surveillance systems.

BACKGROUND

Public health surveillance is the ongoing, systematic collection, analysis, interpretation, and dissemination of data regarding a health-related event for use in public health action to reduce morbidity and mortality and to improve health (*5–7*). Data disseminated by a public health surveillance system can be used for immediate public health action, program planning and evaluation, and formulating research hypotheses. For example, data from a public health surveillance system can be used to

- guide immediate action for cases of public health importance;
- measure the burden of a disease (or other health-related event), including changes in related factors, the identification of populations at high risk, and the identification of new or emerging health concerns;
- monitor trends in the burden of a disease (or other health-related event), including the detection of epidemics (outbreaks) and pandemics;
- guide the planning, implementation, and evaluation of programs to prevent and control disease, injury, or adverse exposure;
- evaluate public policy;
- detect changes in health practices and the effects of these changes;
- prioritize the allocation of health resources;
- describe the clinical course of disease; and
- provide a basis for epidemiologic research.

Public health surveillance activities are generally authorized by legislators and carried out by public health officials. Public health surveillance systems have been developed to address a range of public health needs. In addition, public health information systems have been defined to include a variety of data sources essential to public health action and are often used for surveillance (*8*). These systems vary from a simple system collecting data from a single source, to electronic systems that receive data from many sources in multiple formats, to complex surveys. The number and variety of systems will likely increase with advances in electronic data interchange and integration of data, which will also heighten the importance of patient privacy, data confidentiality, and system security. Appropriate institutions/agencies/scientific officials should be consulted with any projects regarding pubic health surveillance.

Variety might also increase with the range of health-related events under surveillance. In these guidelines, the term "health-related event" refers to any subject related to a public health surveillance system. For example, a health-related event could include infectious, chronic, or zoonotic diseases; injuries; exposures to toxic substances; health promoting or damaging behaviors; and other surveilled events associated with public health action.

The purpose of evaluating public health surveillance systems is to ensure that problems of public health importance are being monitored efficiently and effectively. Public health surveillance systems should be evaluated periodically, and the evaluation should include recommendations for improving quality, efficiency, and usefulness. The goal of these guidelines is to organize the evaluation of a public health surveillance system. Broad topics are outlined into which program-specific qualities can be integrated. Evaluation of a public health surveillance system focuses on how well the system operates to meet its purpose and objectives.

The evaluation of public health surveillance systems should involve an assessment of system attributes, including simplicity, flexibility, data quality, acceptability, sensitivity, predictive value positive, representativeness, timeliness, and stability. With the continuing advancement of technology and the importance of information architecture and related concerns, inherent in these attributes are certain public health informatics concerns for public health surveillance systems. These concerns include comparable hardware and software, standard user interface, standard data format and coding, appropriate quality checks, and adherence to confidentiality and security standards (*9*). Because public health surveillance systems vary in methods, scope, purpose, and objectives, attributes that are important to one system might be less important to another. A public health surveillance system should emphasize those attributes that are most important for the objectives of the system. Efforts to improve certain attributes (e.g., the ability of a public health surveillance system to detect a health-related event [sensitivity]) might detract from other attributes (e.g., simplicity or timeliness). An evaluation of the public health surveillance system must therefore consider those attributes that are of the highest priority for a given system and its objectives. Considering the attributes that are of the highest priority, the guidelines in this report describe many tasks and related activities that can be applied in the evaluation of public health surveillance systems, with the understanding that all activities under the tasks might not be appropriate for all systems.

Organization of This Report

This report begins with descriptions of each of the tasks involved in evaluating a public health surveillance system. These tasks are adapted from the steps in program evaluation in the *Framework for Program Evaluation in Public Health* (*4*) as well as from the elements in the original guidelines for evaluating surveillance systems (*1*). The report concludes with a summary statement regarding evaluating surveillance systems. A checklist that can be detached or photocopied and used when the evaluation is implemented is also included (Appendix A).

To assess the quality of the evaluation activities, relevant standards are provided for each of the tasks for evaluating a public health surveillance system (Appendix B). These standards are adapted from the standards for effective evaluation (i.e., utility, feasibility, propriety, and accuracy) in the *Framework for Program Evaluation in Public Health* (*4*). Because all activities under the evaluation tasks might not be appropriate for all systems, only those standards that are appropriate to an evaluation should be used.

Task A. Engage the Stakeholders in the Evaluation

Stakeholders can provide input to ensure that the evaluation of a public health surveillance system addresses appropriate questions and assesses pertinent attributes and that its findings will be acceptable and useful. In that context, we define stakeholders as those persons or organizations who use data for the promotion of healthy lifestyles and the prevention and control of disease, injury, or adverse exposure. Those stakeholders who might be interested in defining questions to be addressed by the surveillance system evaluation and subsequently using the findings from it are public health practitioners; health-care providers; data providers and users; representatives of affected communities; governments at the local, state, and federal levels; and professional and private nonprofit organizations.

Task B. Describe the Surveillance System to be Evaluated

Activities

- Describe the public health importance of the health-related event under surveillance.
- Describe the purpose and operation of the system.
- Describe the resources used to operate the system.

Discussion

To construct a balanced and reliable description of the system, multiple sources of information might be needed. The description of the system can be improved by consulting with a variety of persons involved with the system and by checking reported descriptions of the system against direct observation.

B.1. Describe the Public Health Importance of the Health-Related Event Under Surveillance

Definition. The public health importance of a health-related event and the need to have that event under surveillance can be described in several ways. Health-related events that affect many persons or that require large expenditures of resources are of public health importance. However, health-related events that affect few persons might also be important, especially if the events cluster in time and place (e.g., a limited outbreak of a severe disease). In other instances, public concerns might focus attention on a particular health-related event, creating or heightening the importance of an evaluation. Diseases that are now rare because of successful control measures might be perceived as unimportant, but their level of importance should be assessed as a possible sentinel health-related event or for their potential to reemerge. Finally, the public health importance of a health-related event is influenced by its level of preventability (*10*).

Measures. Parameters for measuring the importance of a health-related event—and therefore the public health surveillance system with which it is monitored—can include (*7*)

- indices of frequency (e.g., the total number of cases and/or deaths; incidence rates, prevalence, and/or mortality rates); and summary measures of population health status (e.g., quality-adjusted life years [QALYS]);
- indices of severity (e.g., bed-disability days, case-fatality ratio, and hospitalization rates and/or disability rates);
- disparities or inequities associated with the health-related event;
- costs associated with the health-related event;
- preventability (*10*);
- potential clinical course in the absence of an intervention (e.g., vaccinations) (*11,12*); and
- public interest.

Efforts have been made to provide summary measures of population health status that can be used to make comparative assessments of the health needs of populations (*13*). Perhaps the best known of these measures are QALYs, years of healthy life (YHLs), and disability-adjusted life years (DALYs). Based on attributes that represent health status and life expectancy, QALYs, YHLs, and DALYs provide one-dimensional measures of overall health. In addition, attempts have been made to quantify the public health importance of various diseases and other health-related events. In a study that describes such an approach, a score was used that takes into account age-specific morbidity and mortality rates as well as health-care costs (*14*). Another study used a model that ranks public health concerns according to size, urgency, severity of the problem, economic loss, effect on others, effectiveness, propriety, economics, acceptability, legality of solutions, and availability of resources (*15*).

Preventability can be defined at several levels, including primary prevention (preventing the occurrence of disease or other health-related event), secondary prevention (early detection and intervention with the aim of reversing, halting, or at least retarding the progress of a condition), and tertiary prevention (minimizing the effects of disease and disability among persons already ill). For infectious diseases, preventability can also be described as reducing the secondary attack rate or the number of cases transmitted to contacts of the primary case. From the perspective of surveillance, preventability reflects the potential for effective public health intervention at any of these levels.

B.2. Describe the Purpose and Operation of the Surveillance System

Methods. Methods for describing the operation of the public health surveillance system include

- List the purpose and objectives of the system.
- Describe the planned uses of the data from the system.
- Describe the health-related event under surveillance, including the case definition for each specific condition.

- Cite any legal authority for the data collection.
- Describe where in the organization(s) the system resides, including the context (e.g., the political, administrative, geographic, or social climate) in which the system evaluation will be done.
- Describe the level of integration with other systems, if appropriate.
- Draw a flow chart of the system.
- Describe the components of the system. For example
  - — What is the population under surveillance?
  - — What is the period of time of the data collection?
  - — What data are collected and how are they collected?
  - — What are the reporting sources of data for the system?
  - — How are the system's data managed (e.g., the transfer, entry, editing, storage, and back up of data)? Does the system comply with applicable standards for data formats and coding schemes? If not, why?
  - — How are the system's data analyzed and disseminated?
  - — What policies and procedures are in place to ensure patient privacy, data confidentiality, and system security? What is the policy and procedure for releasing data? Do these procedures comply with applicable federal and state statutes and regulations? If not, why?
  - — Does the system comply with an applicable records management program? For example, are the system's records properly archived and/or disposed of?

Discussion. The purpose of the system indicates why the system exists, whereas its objectives relate to how the data are used for public health action. The objectives of a public health surveillance system, for example, might address immediate public health action, program planning and evaluation, and formation of research hypotheses (see Background). The purpose and objectives of the system, including the planned uses of its data, establish a frame of reference for evaluating specific components.

A public health surveillance system is dependent on a clear case definition for the health-related event under surveillance (*7*). The case definition of a health-related event can include clinical manifestations (i.e., symptoms), laboratory results, epidemiologic information (e.g., person, place, and time), and/or specified behaviors, as well as levels of certainty (e.g., confirmed/definite, probable/presumptive, or possible/suspected). The use of a standard case definition increases the specificity of reporting and improves the comparability of the health-related event reported from different sources of data, including geographic areas. Case definitions might exist for a variety of health-related events under surveillance, including diseases, injuries, adverse exposures, and risk factor or protective behaviors. For example, in the United States, CDC and the Council of State and Territorial Epidemiologists (CSTE) have agreed on standard case definitions for selected infectious diseases (*16*). In addition, CSTE publishes Position Papers that discuss and define a variety of health-related events (*17*). When possible, a public health surveillance system should use an established case definition, and if it does not, an explanation should be provided.

The evaluation should assess how well the public health surveillance system is integrated with other surveillance and health information systems (e.g., data exchange and sharing in multiple formats, and transformation of data). Streamlining related systems into an integrated public health surveillance network enables individual systems to meet specific data collection needs while avoiding the duplication of effort and lack of standardization that can arise from independent systems (*18*). An integrated system can address comorbidity concerns (e.g., persons infected with human immunodeficiency virus and *Mycobacterium tuberculosis*); identify previously unrecognized risk factors; and provide the means for monitoring additional outcomes from a health-related event. When CDC's NEDSS is completed, it will electronically integrate and link together several types of surveillance activities and facilitate more accurate and timely reporting of disease information to CDC and state and local health departments (*2*).

CSTE has organized professional discussion among practicing public health epidemiologists at state and federal public health agencies. CSTE has also proposed a national public health surveillance system to serve as a basis for local and state public health agencies to a) prioritize surveillance and health information activities and b) advocate for necessary resources for public health agencies at all levels (*19*). This national public health system would be a conceptual framework and virtual surveillance system that incorporates both existing and new surveillance systems for health-related events and their determinants.

Listing the discrete steps that are taken in processing the health-event reports by the system and then depicting these steps in a flow chart is often useful. An example of a simplified flow chart for a generic public health surveillance system is included in this report (Figure 1). The mandates and business processes of the lead agency that operates the system and the participation of other agencies could be included in this chart. The architecture and data flow of the system can also be depicted in the chart (*20,21*). A chart of architecture and data flow should be sufficiently detailed to explain all of the functions of the system, including average times between steps and data transfers.

The description of the components of the public health surveillance system could include discussions related to public health informatics concerns, including comparable hardware and software, standard user interface, standard data format and coding, appropriate quality checks, and adherence to confidentiality and security standards (*9*). For example, comparable hardware and software, standard user interface, and standard data format and coding facilitate efficient data exchange, and a set of common data elements are important for effectively matching data within the system or to other systems.

To document the information needs of public health, CDC, in collaboration with state and local health departments, is developing the Public Health Conceptual Data Model to a) establish data standards for public health, including data definitions, component structures (e.g., for complex data types), code values, and data use; b) collaborate with national health informatics standard-setting bodies to define standards for the exchange of information among public health agencies and health-care providers; and c) construct computerized information systems that conform to established data and data interchange standards for use in the management of data relevant to public health (*22*). In addition, the description of the system's data management might address who is editing the data, how and at what levels the data are edited, and what checks are in place to ensure data quality.

In response to HIPAA mandates, various standard development organizations and terminology and coding groups are working collaboratively to harmonize their separate systems (*23*). For example, both the Accredited Standards Committee X12 (*24*), which has dealt principally with standards for health insurance transactions, and Health Level Seven (HL7) (*25*), which has dealt with standards for clinical messaging and exchange of clinical information with health-care organizations (e.g., hospitals), have collaborated on a standardized approach for providing supplementary information to support health-care claims (*26*). In the area of classification and coding of diseases and other medical terms, the National Library of Medicine has traditionally provided the Unified Medical Language System, a metathesaurus for clinical coding systems that allows terms in one coding system to be mapped to another (*27*). The passage of

FIGURE 1. Simplified flow chart for a generic surveillance system

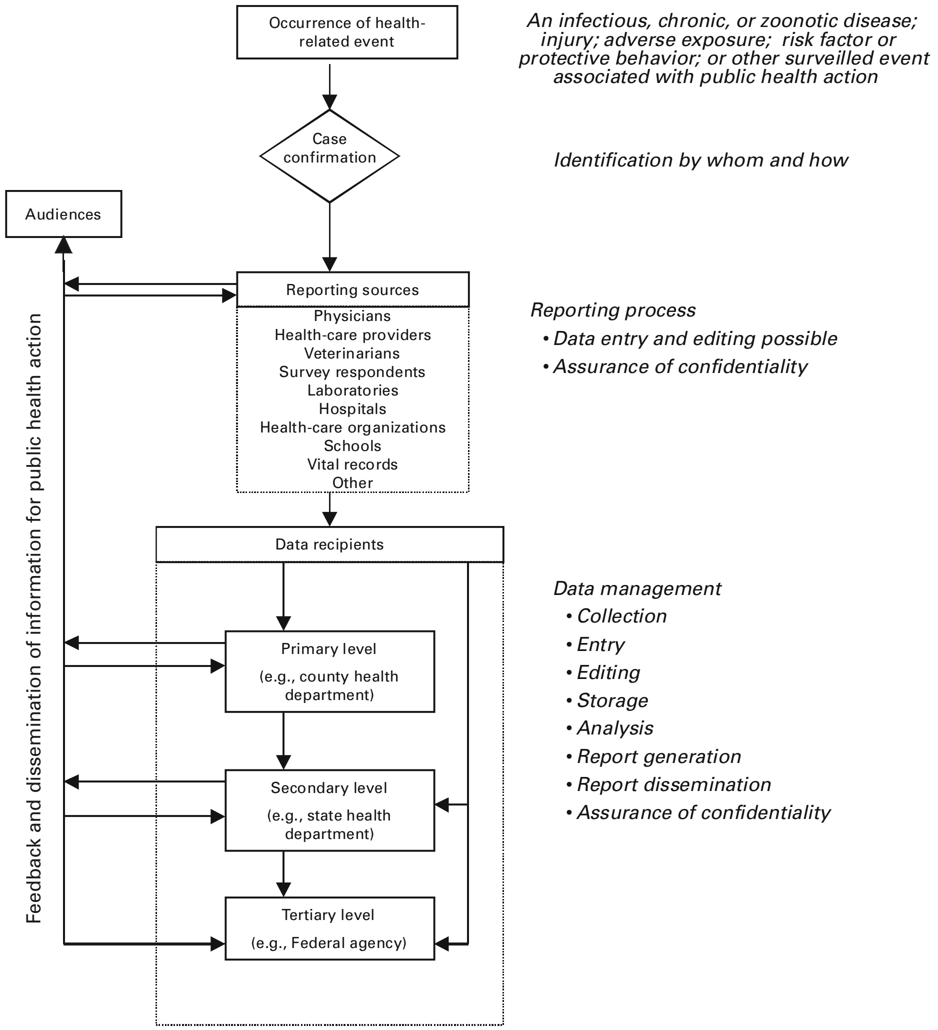

HIPAA and the anticipated adoption of standards for electronic medical records have increased efforts directed toward the integration of clinical terminologies (*23*) (e.g., the merge of the College of American Pathologists' Systematized Nomenclature of Medicine [SNOMED®] [*28*] and the British Read Codes, the National Health Service thesaurus of health-care terms in Great Britain).

The data analysis description might indicate who analyzes the data, how they are analyzed, and how often. This description could also address how the system ensures that appropriate scientific methods are used to analyze the data.

The public health surveillance system should operate in a manner that allows effective dissemination of health data so that decision makers at all levels can readily understand the implications of the information (*7*). Options for disseminating data and/or information from the system include electronic data interchange; public-use data files; the Internet; press releases; newsletters; bulletins; annual and other types of reports; publication in scientific, peer-reviewed journals; and poster and oral presentations, including those at individual, community, and professional meetings. The audiences for health data and information can include public health practitioners, health-care providers, members of affected communities, professional and voluntary organizations, policymakers, the press, and the general public.

In conducting surveillance, public health agencies are authorized to collect personal health data about persons and thus have an obligation to protect against inappropriate use or release of that data. The protection of patient privacy (recognition of a person's right not to share information about him or herself), data confidentiality (assurance of authorized data sharing), and system security (assurance of authorized system access) is essential to maintaining the credibility of any surveillance system. This protection must ensure that data in a surveillance system regarding a person's health status are shared only with authorized persons. Physical, administrative, operational, and computer safeguards for securing the system and protecting its data must allow authorized access while denying access by unauthorized users.

A related concern in protecting health data is data release, including procedures for releasing record-level data; aggregate tabular data; and data in computer-based, interactive query systems. Even though personal identifiers are removed before data are released, the removal of these identifiers might not be a sufficient safeguard for sharing health data. For example, the inclusion of demographic information in a line-listed data file for a small number of cases could lead to indirect identification of a person even though personal identifiers were not provided. In the United States, CDC and CSTE have negotiated a policy for the release of data from the National Notifiable Disease Surveillance System (*29*) to facilitate its use for public health while preserving the confidentiality of the data (*30*). The policy is being evaluated for revision by CDC and CSTE.

Standards for the privacy of individually identifiable health data have been proposed in response to HIPAA (*3*). A model state law has been composed to address privacy, confidentiality, and security concerns arising from the acquisition, use, disclosure, and storage of health information by public health agencies at the state and local levels (*31*). In addition, the Federal Committee on Statistical Methodology's series of *Statistical Policy Working Papers* includes reviews of statistical methods used by federal agencies and their contractors that release statistical tables or microdata files

that are collected from persons, businesses, or other units under a pledge of confidentiality. These working papers contain basic statistical methods to limit disclosure (e.g., rules for data suppression to protect privacy and to minimize mistaken inferences from small numbers) and provide recommendations for improving disclosure limitation practices (*32*).

A public health surveillance system might be legally required to participate in a records management program. Records can consist of a variety of materials (e.g., completed forms, electronic files, documents, and reports) that are connected with operating the surveillance system. The proper management of these records prevents a "loss of memory" or "cluttered memory" for the agency that operates the system, and enhances the system's ability to meet its objectives.

B.3. Describe the Resources Used to Operate the Surveillance System

Definition. In this report, the methods for assessing resources cover only those resources directly required to operate a public health surveillance system. These resources are sometimes referred to as "direct costs" and include the personnel and financial resources expended in operating the system.

Methods. In describing these resources consider the following:

- **Funding source(s):** Specify the source of funding for the surveillance system. In the United States, public health surveillance often results from a collaboration among federal, state, and local governments.
- **Personnel requirements:** Estimate the time it takes to operate the system, including the collection, editing, analysis, and dissemination of data (e.g., person-time expended per year of operation). These measures can be converted to dollar estimates by multiplying the person-time by appropriate salary and benefit costs.
- **Other resources:** Determine the cost of other resources, including travel, training, supplies, computer and other equipment, and related services (e.g., mail, telephone, computer support, Internet connections, laboratory support, and hardware and software maintenance).

When appropriate, the description of the system's resources should consider all levels of the public health system, from the local health-care provider to municipal, county, state, and federal health agencies. Resource estimation for public health surveillance systems have been implemented in Vermont (Table 1) and Kentucky (Table 2).

Resource Estimation in Vermont. Two methods of collecting public health surveillance data in Vermont were compared (*33*). The passive system was already in place and consisted of unsolicited reports of notifiable diseases to the district offices or state health department. The active system was implemented in a probability sample of physician practices. Each week, a health department employee called these practitioners to solicit reports of selected notifiable diseases.

In comparing the two systems, an attempt was made to estimate their costs. The estimates of direct expenses were computed for the public health surveillance systems (Table 1).

Resource Estimation in Kentucky. Another example of resource estimation was provided by an assessment of the costs of a public health surveillance system involving the active solicitation of case reports of type A hepatitis in Kentucky (Table 2) (*34*). The resources that were invested into the direct operation of the system in 1983 were for

TABLE 1. Comparison of estimated expenses for health department active and passive surveillance systems — Vermont, June 1, 1980–May 31, 1981*

Expenses	Surveillance system: Active[†]	Passive[§]
Paper	$114	$80
Mailing	185	48
Telephone	1,947	175
Personnel		
Secretary	3,000	2,000
Public health nurse	14,025	0
**Total**	**$19,271**	**$2,303**

*Vogt RL, LaRue D, Klaucke DN, Jillson DA. Comparison of an active and passive surveillance system of primary care providers for hepatitis, measles, rubella, and salmonellosis in Vermont. Am J Public Health 1983;73:795–7.

[†] Active surveillance — weekly calls were made from health departments requesting reports.

[§] Passive surveillance — provider-initiated reporting.

personnel and telephone expenses and were estimated at $3,764 and $535, respectively. Nine more cases were found through this system than would have been found through the passive surveillance system, and an estimated seven hepatitis cases were prevented through administering prophylaxis to the contacts of the nine case-patients.

Discussion. This approach to assessing resources includes only those personnel and material resources required for the operation of surveillance and excludes a broader definition of costs that might be considered in a more comprehensive evaluation. For example, the assessment of resources could include the estimation of indirect costs (e.g., follow-up laboratory tests) and costs of secondary data sources (e.g., vital statistics or survey data).

The assessment of the system's operational resources should not be done in isolation of the program or initiative that relies on the public health surveillance system. A more formal economic evaluation of the system (i.e., judging costs relative to benefits) could be included with the resource description. Estimating the effect of the system on decision making, treatment, care, prevention, education, and/or research might be possible (*35,36*). For some surveillance systems, however, a more realistic approach would be to judge costs based on the objectives and usefulness of the system.

Task C. Focus the Evaluation Design

Definition

The direction and process of the evaluation must be focused to ensure that time and resources are used as efficiently as possible.

Methods

Focusing the evaluation design for a public health surveillance system involves

- determining the specific purpose of the evaluation (e.g., a change in practice);
- identifying stakeholders (Task A) who will receive the findings and recommendations of the evaluation (i.e., the intended users);

TABLE 2. Costs of a 22-week active surveillance program for hepatitis A — Kentucky, 1983*

Activity	Estimated costs
**Central office**	
Surveillance	
Personnel	$3,764
Telephone	535
**Local health offices**[†]	
Contact tracing	
Personnel	647
Telephone	149
Travel	31
Contact prophylaxis	
Personnel	469
Immune serum globulin	21
**Total**	**$5,616**

* Hinds MW, Skaggs JW, Bergeisen GH. Benefit-cost analysis of active surveillance of primary care physicians for hepatitis A. Am J Public Health 1985;75:176–7.

† Costs of tracing and providing prophylaxis to 38 additional active surveillance-associated contacts of persons with hepatitis A.

- considering what will be done with the information generated from the evaluation (i.e., the intended uses);
- specifying the questions that will be answered by the evaluation; and
- determining standards for assessing the performance of the system.

Discussion

Depending on the specific purpose of the evaluation, its design could be straightforward or complex. An effective evaluation design is contingent upon a) its specific purpose being understood by all of the stakeholders in the evaluation and b) persons who need to know the findings and recommendations of the design being committed to using the information generated from it. In addition, when multiple stakeholders are involved, agreements that clarify roles and responsibilities might need to be established among those who are implementing the evaluation.

Standards for assessing how the public health surveillance system performs establish what the system must accomplish to be considered successful in meeting its objectives. These standards specify, for example, what levels of usefulness and simplicity are relevant for the system, given its objectives. Approaches to setting useful standards for assessing the system's performance include a review of current scientific literature on the health-related event under surveillance and/or consultation with appropriate specialists, including users of the data.

Task D. Gather Credible Evidence Regarding the Performance of the Surveillance System

Activities

- Indicate the level of usefulness by describing the actions taken as a result of analysis and interpretation of the data from the public health surveillance system. Characterize the entities that have used the data to make decisions and take actions. List other anticipated uses of the data.
- Describe each of the following system attributes:

— Simplicity

— Flexibility

— Data quality

— Acceptability

— Sensitivity

— Predictive value positive

— Representativeness

— Timeliness

— Stability

Discussion

Public health informatics concerns for public health surveillance systems (see Task B.2, Discussion) can be addressed in the evidence gathered regarding the performance of the system. Evidence of the system's performance must be viewed as credible. For example, the gathered evidence must be reliable, valid, and informative for its intended use. Many potential sources of evidence regarding the system's performance exist, including consultations with physicians, epidemiologists, statisticians, behavioral scientists, public health practitioners, laboratory directors, program managers, data providers, and data users.

D.1. Indicate the Level of Usefulness

Definition. A public health surveillance system is useful if it contributes to the prevention and control of adverse health-related events, including an improved understanding of the public health implications of such events. A public health surveillance system can also be useful if it helps to determine that an adverse health-related event previously thought to be unimportant is actually important. In addition, data from a surveillance system can be useful in contributing to performance measures (*37*), including health indicators (*38*) that are used in needs assessments and accountability systems.

Methods. An assessment of the usefulness of a public health surveillance system should begin with a review of the objectives of the system and should consider the system's effect on policy decisions and disease-control programs. Depending on the objectives of a particular surveillance system, the system might be considered useful if it satisfactorily addresses at least one of the following questions. Does the system

- detect diseases, injuries, or adverse or protective exposures of public importance in a timely way to permit accurate diagnosis or identification, prevention or treatment, and handling of contacts when appropriate?
- provide estimates of the magnitude of morbidity and mortality related to the health-related event under surveillance, including the identification of factors associated with the event?
- detect trends that signal changes in the occurrence of disease, injury, or adverse or protective exposure, including detection of epidemics (or outbreaks)?
- permit assessment of the effect of prevention and control programs?
- lead to improved clinical, behavioral, social, policy, or environmental practices? or
- stimulate research intended to lead to prevention or control?

A survey of persons who use data from the system might be helpful in gathering evidence regarding the usefulness of the system. The survey could be done either formally with standard methodology or informally.

Discussion. Usefulness might be affected by all the attributes of a public health surveillance system (see Task D.2, Describe Each System Attribute). For example, increased sensitivity might afford a greater opportunity for identifying outbreaks and understanding the natural course of an adverse health-related event in the population under surveillance. Improved timeliness allows control and prevention activities to be initiated earlier. Increased predictive value positive enables public health officials to more accurately focus resources for control and prevention measures. A representative surveillance system will better characterize the epidemiologic characteristics of a health-related event in a defined population. Public health surveillance systems that are simple, flexible, acceptable, and stable will likely be more complete and useful for public health action.

D.2. Describe Each System Attribute

D.2.a. Simplicity

Definition. The simplicity of a public health surveillance system refers to both its structure and ease of operation. Surveillance systems should be as simple as possible while still meeting their objectives.

Methods. A chart describing the flow of data and the lines of response in a surveillance system can help assess the simplicity or complexity of a surveillance system. A simplified flow chart for a generic surveillance system is included in this report (Figure 1).

The following measures (see Task B.2) might be considered in evaluating the simplicity of a system:

- amount and type of data necessary to establish that the health-related event has occurred (i.e., the case definition has been met);
- amount and type of other data on cases (e.g., demographic, behavioral, and exposure information for the health-related event);
- number of organizations involved in receiving case reports;
- level of integration with other systems;
- method of collecting the data, including number and types of reporting sources, and time spent on collecting data;
- amount of follow-up that is necessary to update data on the case;
- method of managing the data, including time spent on transferring, entering, editing, storing, and backing up data;
- methods for analyzing and disseminating the data, including time spent on preparing the data for dissemination;
- staff training requirements; and
- time spent on maintaining the system.

Discussion. Thinking of the simplicity of a public health surveillance system from the design perspective might be useful. An example of a system that is simple in design is one with a case definition that is easy to apply (i.e., the case is easily ascertained) and in which the person identifying the case will also be the one analyzing and using the information. A more complex system might involve some of the following:

- special or follow-up laboratory tests to confirm the case;
- investigation of the case, including telephone contact or a home visit by public health personnel to collect detailed information;
- multiple levels of reporting (e.g., with the National Notifiable Diseases Surveillance System, case reports might start with the health-care provider who makes the diagnosis and pass through county and state health departments before going to CDC [*29*]); and
- integration of related systems whereby special training is required to collect and/or interpret data.

Simplicity is closely related to acceptance and timeliness. Simplicity also affects the amount of resources required to operate the system.

D.2.b. Flexibility

Definition. A flexible public health surveillance system can adapt to changing information needs or operating conditions with little additional time, personnel, or allocated funds. Flexible systems can accommodate, for example, new health-related events, changes in case definitions or technology, and variations in funding or reporting sources. In addition, systems that use standard data formats (e.g., in electronic data interchange) can be easily integrated with other systems and thus might be considered flexible.

Methods. Flexibility is probably best evaluated retrospectively by observing how a system has responded to a new demand. An important characteristic of CDC's Behavioral Risk Factor Surveillance System (BRFSS) is its flexibility (*39*). Conducted in collaboration with state health departments, BRFSS is an ongoing sample survey that gathers and reports state-level prevalence data on health behaviors related to the leading preventable causes of death as well as data on preventive health practices. The system permits states to add questions of their own design to the BRFSS questionnaire but is uniform enough to allow state-to-state comparisons for certain questions. These state-specific questions can address emergent and locally important health concerns. In addition, states can stratify their BRFSS samples to estimate prevalence data for regions or counties within their respective states.

Discussion. Unless efforts have been made to adapt the public health surveillance system to another disease (or other health-related event), a revised case definition, additional data sources, new information technology, or changes in funding, assessing the flexibility of that system might be difficult. In the absence of practical experience, the design and workings of a system can be examined. Simpler systems might be more flexible (i.e., fewer components will need to be modified when adapting the system for a change in information needs or operating conditions).

D.2.c. Data Quality

Definition. Data quality reflects the completeness and validity of the data recorded in the public health surveillance system.

Methods. Examining the percentage of "unknown" or "blank" responses to items on surveillance forms is a straightforward and easy measure of data quality. Data of high quality will have low percentages of such responses. However, a full assessment of the completeness and validity of the system's data might require a special study. Data values recorded in the surveillance system can be compared to "true" values through, for example, a review of sampled data (*40*), a special record linkage (*41*), or patient interview (*42*). In addition, the calculation of sensitivity (Task D.2.e) and predictive value positive (Task D.2.f) for the system's data fields might be useful in assessing data quality.

Quality of data is influenced by the performance of the screening and diagnostic tests (i.e., the case definition) for the health-related event, the clarity of hardcopy or electronic surveillance forms, the quality of training and supervision of persons who complete these surveillance forms, and the care exercised in data management. A review of these facets of a public health surveillance system provides an indirect measure of data quality.

Discussion. Most surveillance systems rely on more than simple case counts. Data commonly collected include the demographic characteristics of affected persons, details about the health-related event, and the presence or absence of potential risk factors. The quality of these data depends on their completeness and validity.

The acceptability (see Task D.2.d) and representativeness (Task D.2.g) of a public health surveillance system are related to data quality. With data of high quality, the system can be accepted by those who participate in it. In addition, the system can accurately represent the health-related event under surveillance.

D.2.d. Acceptability

Definition. Acceptability reflects the willingness of persons and organizations to participate in the surveillance system.

Methods. Acceptability refers to the willingness of persons in the sponsoring agency that operates the system and persons outside the sponsoring agency (e.g., persons who are asked to report data) to use the system. To assess acceptability, the points of interaction between the system and its participants must be considered (Figure 1), including persons with the health-related event and those reporting cases.

Quantitative measures of acceptability can include

- subject or agency participation rate (if it is high, how quickly it was achieved);
- interview completion rates and question refusal rates (if the system involves interviews);
- completeness of report forms;
- physician, laboratory, or hospital/facility reporting rate; and
- timeliness of data reporting.

Some of these measures might be obtained from a review of surveillance report forms, whereas others would require special studies or surveys.

Discussion. Acceptability is a largely subjective attribute that encompasses the willingness of persons on whom the public health surveillance system depends to provide accurate, consistent, complete, and timely data. Some factors influencing the acceptability of a particular system are

- the public health importance of the health-related event;
- acknowledgment by the system of the person's contribution;
- dissemination of aggregate data back to reporting sources and interested parties;
- responsiveness of the system to suggestions or comments;
- burden on time relative to available time;
- ease and cost of data reporting;
- federal and state statutory assurance of privacy and confidentiality;
- the ability of the system to protect privacy and confidentiality;
- federal and state statute requirements for data collection and case reporting; and
- participation from the community in which the system operates.

D.2.e. Sensitivity

Definition. The sensitivity of a surveillance system can be considered on two levels. First, at the level of case reporting, sensitivity refers to the proportion of cases of a disease (or other health-related event) detected by the surveillance system (*43*). Second, sensitivity can refer to the ability to detect outbreaks, including the ability to monitor changes in the number of cases over time.

Methods. The measurement of the sensitivity of a public health surveillance system is affected by the likelihood that

- certain diseases or other health-related events are occurring in the population under surveillance;
- cases of certain health-related events are under medical care, receive laboratory testing, or are otherwise coming to the attention of institutions subject to reporting requirements;
- the health-related events will be diagnosed/identified, reflecting the skill of health-care providers and the sensitivity of screening and diagnostic tests (i.e., the case definition); and
- the case will be reported to the system.

These situations can be extended by analogy to public health surveillance systems that do not fit the traditional disease care-provider model. For example, the sensitivity of a telephone-based surveillance system of morbidity or risk factors is affected by

- the number of persons who have telephones, who are at home when the call is placed, and who agree to participate;
- the ability of persons to understand the questions and correctly identify their status; and
- the willingness of respondents to report their status.

The extent to which these situations are explored depends on the system and on the resources available for assessing sensitivity. The primary emphasis in assessing sensitivity — assuming that most reported cases are correctly classified — is to estimate the proportion of the total number of cases in the population under surveillance being detected by the system, represented by A/(A+C) in this report (Table 3).

Surveillance of vaccine-preventable diseases provides an example of where the detection of outbreaks is a critical concern (*44*). Approaches that have been recommended for improving sensitivity of reporting vaccine-preventable diseases might be

TABLE 3. Calculation of sensitivity* and predictive value positive† for a surveillance system

Detected by surveillance	Condition present: Yes	Condition present: No	
Yes	True positive A	False positive B	A+B
No	False negative C	True negative D	C+D
	A+C	B+D	Total

* Sensitivity = A/(A+C)

† Predictive value positive (PVP) = A/(A+B)

applicable to other health-related events (*44*). For example, the sensitivity of a system might be improved by

- conducting active surveillance (i.e., contacting all providers and institutions responsible for reporting cases);
- using external standards (or other surveillance indicators) to monitor the quality of case reporting;
- identifying imported cases;
- tracking the number of cases of suspected disease that are reported, investigated, and ruled out as cases;
- monitoring the diagnostic effort (e.g., tracking submission of laboratory requests for diagnostic testing); and
- monitoring the circulation of the agent (e.g., virus or bacterium) that causes the disease.

The capacity for a public health surveillance system to detect outbreaks (or other changes in incidence and prevalence) might be enhanced substantially if detailed diagnostic tests are included in the system. For example, the use of molecular subtyping in the surveillance of *Escherichia coli* O157:H7 infections in Minnesota enabled the surveillance system to detect outbreaks that would otherwise have gone unrecognized (*45*).

The measurement of the sensitivity of the surveillance system (Table 3) requires a) collection of or access to data usually external to the system to determine the true frequency of the condition in the population under surveillance (*46*) and b) validation of the data collected by the system. Examples of data sources used to assess the sensitivity of health information or public health surveillance systems include medical records (*47,48*) and registries (*49,50*). In addition, sensitivity can be assessed through estimations of the total cases in the population under surveillance by using capture-recapture techniques (*51,52*).

To adequately assess the sensitivity of the public health surveillance system, calculating more than one measurement of the attribute might be necessary. For example, sensitivity could be determined for the system's data fields, for each data source or for combinations of data sources (*48*), for specific conditions under surveillance (*53*), or for each of several years (*54*). The use of a Venn diagram might help depict measurements of sensitivity for combinations of the system's data sources (*55*).

Discussion. A literature review can be helpful in determining sensitivity measurements for a public health surveillance system (*56*). The assessment of the sensitivity of each data source, including combinations of data sources, can determine if the elimination of a current data source or if the addition of a new data source would affect the overall surveillance results (*48*).

A public health surveillance system that does not have high sensitivity can still be useful in monitoring trends as long as the sensitivity remains reasonably constant over time. Questions concerning sensitivity in surveillance systems most commonly arise when changes in the occurrence of a health-related event are noted. Changes in sensitivity can be precipitated by some circumstances (e.g., heightened awareness of a health-related event, introduction of new diagnostic tests, and changes in the method of conducting surveillance). A search for such "artifacts" is often an initial step in outbreak investigations.

D.2.f. Predictive Value Positive

Definition. Predictive value positive (PVP) is the proportion of reported cases that actually have the health-related event under surveillance (*43*).

Methods. The assessment of sensitivity and of PVP provide different perspectives regarding how well the system is operating. Depending on the objectives of the public health surveillance system, assessing PVP whenever sensitivity has been assessed might be necessary (*47–50,53*). In this report, PVP is represented by A/(A+B) (Table 3).

In assessing PVP, primary emphasis is placed on the confirmation of cases reported through the surveillance system. The effect of PVP on the use of public health resources can be considered on two levels. At the level of case detection, PVP affects the amount of resources used for case investigations. For example, in some states, every reported case of type A hepatitis is promptly investigated by a public health nurse, and contacts at risk are referred for prophylactic treatment. A surveillance system with low PVP, and therefore frequent “false-positive” case reports, would lead to misdirected resources.

At the level of outbreak (or epidemic) detection, a high rate of erroneous case reports might trigger an inappropriate outbreak investigation. Therefore, the proportion of epidemics identified by the surveillance system that are true epidemics can be used to assess this attribute.

Calculating the PVP might require that records be kept of investigations prompted by information obtained from the public health surveillance system. At the level of case detection, a record of the number of case investigations completed and the proportion of reported persons who actually had the health-related event under surveillance would allow the calculation of the PVP. At the level of outbreak detection, the review of personnel activity reports, travel records, and telephone logbooks might enable the assessment of PVP. For some surveillance systems, however, a review of data external to the system (e.g., medical records) might be necessary to confirm cases to calculate PVP. Examples of data sources used to assess the PVP of health information or public health surveillance systems include medical records (*48,57*), registries (*49,58*), and death certificates (*59*).

To assess the PVP of the system adequately, calculating more than one measurement of the attribute might be necessary. For example, PVP could be determined for the system’s data fields, for each data source or combinations of data sources (*48*), or for specific health-related events (*49*).

Discussion. PVP is important because a low value means that noncases might be investigated, and outbreaks might be identified that are not true but are instead artifacts of the public health surveillance system (e.g., a “pseudo-outbreak”). False-positive reports can lead to unnecessary interventions, and falsely detected outbreaks can lead to costly investigations and undue concern in the population under surveillance. A public health surveillance system with a high PVP will lead to fewer misdirected resources.

The PVP reflects the sensitivity and specificity of the case definition (i.e., the screening and diagnostic tests for the health-related event) and the prevalence of the health-related event in the population under surveillance. The PVP can improve with increasing specificity of the case definition. In addition, good communication between the persons who report cases and the receiving agency can lead to an improved PVP.

D.2.g. Representativeness

Definition. A public health surveillance system that is representative accurately describes the occurrence of a health-related event over time and its distribution in the population by place and person.

Methods. Representativeness is assessed by comparing the characteristics of reported events to all such actual events. Although the latter information is generally not known, some judgment of the representativeness of surveillance data is possible, based on knowledge of

- characteristics of the population, including, age, socioeconomic status, access to health care, and geographic location (*60*);
- clinical course of the disease or other health-related event (e.g., latency period, mode of transmission, and outcome [e.g., death, hospitalization, or disability]);
- prevailing medical practices (e.g., sites performing diagnostic tests and physician-referral patterns) (*33,61*); and
- multiple sources of data (e.g., mortality rates for comparison with incidence data and laboratory reports for comparison with physician reports).

Representativeness can be examined through special studies that seek to identify a sample of all cases. For example, the representativeness of a regional injury surveillance system was examined using a systematic sample of injured persons (*62*). The study examined statistical measures of population variables (e.g., age, sex, residence, nature of injury, and hospital admission) and concluded that the differences in the distribution of injuries in the system's database and their distribution in the sampled data should not affect the ability of the surveillance system to achieve its objectives.

For many health-related events under surveillance, the proper analysis and interpretation of the data require the calculation of rates. The denominators for these rate calculations are often obtained from a completely separate data system maintained by another agency (e.g., the United States Bureau of the Census in collaboration with state governments [*63*]). The choice of an appropriate denominator for the rate calculation should be given careful consideration to ensure an accurate representation of the health-related event over time and by place and person. For example, numerators and denominators must be comparable across categories (e.g., race [*64*], age, residence, and/or time period), and the source for the denominator should be consistent over time when measuring trends in rates. In addition, consideration should be given to the selection of the standard population for the adjustment of rates (*65*).

Discussion. To generalize findings from surveillance data to the population at large, the data from a public health surveillance system should accurately reflect the characteristics of the health-related event under surveillance. These characteristics generally relate to time, place, and person. An important result of evaluating the representativeness of a surveillance system is the identification of population subgroups that might be systematically excluded from the reporting system through inadequate methods of monitoring them. This evaluation process enables appropriate modification of data collection procedures and more accurate projection of incidence of the health-related event in the target population (*66*).

For certain health-related events, the accurate description of the event over time involves targeting appropriate points in a broad spectrum of exposure and the resultant disease or condition. In the surveillance of cardiovascular diseases, for example, it might be useful to distinguish between preexposure conditions (e.g., tobacco use policies and social norms), the exposure (e.g., tobacco use, diet, exercise, stress, and genetics), a pre-symptomatic phase (e.g., cholesterol and homocysteine levels), early-staged disease (e.g., abnormal stress test), late-staged disease (e.g., angina and acute

myocardial infarction), and death from the disease. The measurement of risk factor behaviors (e.g., tobacco use) might enable the monitoring of important aspects in the development of a disease or other health-related event.

Because surveillance data are used to identify groups at high risk and to target and evaluate interventions, being aware of the strengths and limitations of the system's data is important. Errors and bias can be introduced into the system at any stage (*67*). For example, case ascertainment (or selection) bias can result from changes in reporting practices over time or from differences in reporting practices by geographic location or by health-care providers. Differential reporting among population subgroups can result in misleading conclusions about the health-related event under surveillance.

D.2.h. Timeliness

Definition. Timeliness reflects the speed between steps in a public health surveillance system.

Methods. A simplified example of the steps in a public health surveillance system is included in this report (Figure 2). The time interval linking any two of these steps can be examined. The interval usually considered first is the amount of time between the onset of a health-related event and the reporting of that event to the public health agency responsible for instituting control and prevention measures. Factors affecting the time involved during this interval can include the patient's recognition of symptoms, the patient's acquisition of medical care, the attending physician's diagnosis or

FIGURE 2. Simplified example of steps in a surveillance system

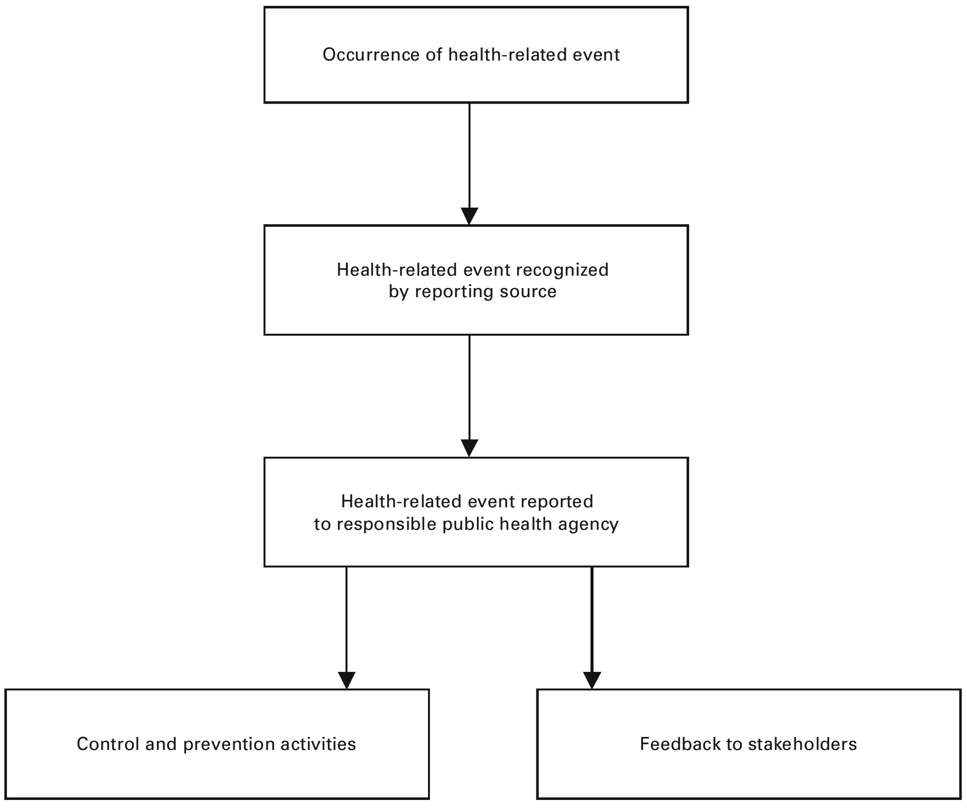

submission of a laboratory test, the laboratory reporting test results back to the physician and/or to a public health agency, and the physician reporting the event to a public health agency. Another aspect of timeliness is the time required for the identification of trends, outbreaks, or the effect of control and prevention measures. Factors that influence the identification process can include the severity and communicability of the health-related event, staffing of the responsible public health agency, and communication among involved health agencies and organizations. The most relevant time interval might vary with the type of health-related event under surveillance. With acute or infectious diseases, for example, the interval from the onset of symptoms or the date of exposure might be used. With chronic diseases, it might be more useful to look at elapsed time from diagnosis rather than from the date of symptom onset.

Discussion. The timeliness of a public health surveillance system should be evaluated in terms of availability of information for control of a health-related event, including immediate control efforts, prevention of continued exposure, or program planning. The need for rapidity of response in a surveillance system depends on the nature of the health-related event under surveillance and the objectives of that system. A study of a public health surveillance system for *Shigella* infections, for example, indicated that the typical case of shigellosis was brought to the attention of health officials 11 days after onset of symptoms — a period sufficient for the occurrence of secondary and tertiary transmission. This example indicates that the level of timeliness was not satisfactory for effective disease control (*68*). However, when a long period of latency occurs between exposure and appearance of disease, the rapid identification of cases of illness might not be as important as the rapid availability of exposure data to provide a basis for interrupting and preventing exposures that lead to disease. For example, children with elevated blood lead levels and no clinically apparent illness are at risk for adverse health-related events. CDC recommends that follow-up of asymptomatic children with elevated blood lead levels include educational activities regarding lead poisoning prevention and investigation and remediation of sources of lead exposure (*69*). In addition, surveillance data are being used by public health agencies to track progress toward national and state health objectives (*38,70*).

The increasing use of electronic data collection from reporting sources (e.g., an electronic laboratory-based surveillance system) and via the Internet (a web-based system), as well as the increasing use of electronic data interchange by surveillance systems, might promote timeliness (*6,29,71,72*).

D.2.i. Stability

Definition. Stability refers to the reliability (i.e., the ability to collect, manage, and provide data properly without failure) and availability (the ability to be operational when it is needed) of the public health surveillance system.

Methods. Measures of the system's stability can include

- the number of unscheduled outages and down times for the system's computer;
- the costs involved with any repair of the system's computer, including parts, service, and amount of time required for the repair;
- the percentage of time the system is operating fully;
- the desired and actual amount of time required for the system to collect or receive data;

- the desired and actual amount of time required for the system to manage the data, including transfer, entry, editing, storage, and back-up of data; and
- the desired and actual amount of time required for the system to release data.

Discussion. A lack of dedicated resources might affect the stability of a public health surveillance system. For example, workforce shortages can threaten reliability and availability. Yet, regardless of the health-related event being monitored, a stable performance is crucial to the viability of the surveillance system. Unreliable and unavailable surveillance systems can delay or prevent necessary public health action.

A more formal assessment of the system's stability could be made through modeling procedures (*73*). However, a more useful approach might involve assessing stability based on the purpose and objectives of the system.

Task E. Justify and State Conclusions, and Make Recommendations

Conclusions from the evaluation can be justified through appropriate analysis, synthesis, interpretation, and judgement of the gathered evidence regarding the performance of the public health surveillance system (Task D). Because the stakeholders (Task A) must agree that the conclusions are justified before they will use findings from the evaluation with confidence, the gathered evidence should be linked to their relevant standards for assessing the system's performance (Task C). In addition, the conclusions should state whether the surveillance system is addressing an important public health problem (Task B.1) and is meeting its objectives (Task B.2).

Recommendations should address the modification and/or continuation of the public health surveillance system. Before recommending modifications to a system, the evaluation should consider the interdependence of the system's costs (Task B.3) and attributes (Task D.2). Strengthening one system attribute could adversely affect another attribute of a higher priority. Efforts to improve sensitivity, PVP, representativeness, timeliness, and stability can increase the cost of a surveillance system, although savings in efficiency with computer technology (e.g., electronic reporting) might offset some of these costs. As sensitivity and PVP approach 100%, a surveillance system is more likely to be representative of the population with the event under surveillance. However, as sensitivity increases, PVP might decrease. Efforts to increase sensitivity and PVP might increase the complexity of a surveillance system — potentially decreasing its acceptability, timeliness, and flexibility. In a study comparing health-department–initiated (active) surveillance and provider-initiated (passive) surveillance, for example, the active surveillance did not improve timeliness, despite increased sensitivity (*61*). In addition, the recommendations can address concerns about ethical obligations in operating the system (*74*).

In some instances, conclusions from the evaluation indicate that the most appropriate recommendation is to discontinue the public health surveillance system; however, this type of recommendation should be considered carefully before it is issued. The cost of renewing a system that has been discontinued could be substantially greater than the cost of maintaining it. The stakeholders in the evaluation should consider relevant public health and other consequences of discontinuing a surveillance system.

Task F. Ensure Use of Evaluation Findings and Share Lessons Learned

Deliberate effort is needed to ensure that the findings from a public health surveillance system evaluation are used and disseminated appropriately. When the evaluation design is focused (Task C), the stakeholders (Task A) can comment on decisions that might affect the likelihood of gathering credible evidence regarding the system's performance. During the implementation of the evaluation (Tasks D and E), considering how potential findings (particularly negative findings) could affect decisions made about the surveillance system might be necessary. When conclusions from the evaluation and recommendations are made (Task E), follow-up might be necessary to remind intended users of their planned uses and to prevent lessons learned from becoming lost or ignored.

Strategies for communicating the findings from the evaluation and recommendations should be tailored to relevant audiences, including persons who provided data used for the evaluation. In the public health community, for example, a formal written report or oral presentation might be important but not necessarily the only means of communicating findings and recommendations from the evaluation to relevant audiences. Several examples of formal written reports of surveillance evaluations have been included in peer-reviewed journals (*51,53,57,59,75*).

SUMMARY

The guidelines in this report address evaluations of public health surveillance systems. However, these guidelines could also be applied to several systems, including health information systems used for public health action, surveillance systems that are pilot tested, and information systems at individual hospitals or health-care centers. Additional information can also be useful for planning, establishing, as well as efficiently and effectively monitoring a public health surveillance system (*6–7*).

To promote the best use of public health resources, all public health surveillance systems should be evaluated periodically. No perfect system exists; however, and trade-offs must always be made. Each system is unique and must balance benefit versus personnel, resources, and cost allocated to each of its components if the system is to achieve its intended purpose and objectives.

The appropriate evaluation of public health surveillance systems becomes paramount as these systems adapt to revised case definitions, new health-related events, new information technology (including standards for data collection and sharing), current requirements for protecting patient privacy, data confidentiality, and system security. The goal of this report has been to make the evaluation process inclusive, explicit, and objective. Yet, this report has presented guidelines — not absolutes — for the evaluation of public health surveillance systems. Progress in surveillance theory, technology, and practice continues to occur, and guidelines for evaluating a surveillance system will necessarily evolve.

References

1. CDC. Guidelines for evaluating surveillance systems. MMWR 1988;37(No. S-5).
2. Health Information and Surveillance System Board. Integration Project: National Electronic Disease Surveillance System. Available at <http://www.cdc.gov/od/hissb/act_int.htm>. Accessed May 7, 2001.

3. Department of Health and Human Services. Administrative simplification. Available at <http://aspe.os.dhhs.gov/admnsimp/Index.htm>. Accessed May 7, 2001.
4. CDC. Framework for program evaluation in public health. MMWR 1999;48(RR-11).
5. Thacker SB. Historical development. In: Teutsch SM, Churchill RE, eds. Principles and practice of public health surveillance, 2nd ed. New York, NY: Oxford University Press, 2000.
6. Buehler JW. Surveillance. In: Rothman KJ, Greenland S. Modern epidemiology, 2nd ed. Philadelphia, PA: Lippencott-Raven, 1998.
7. Teutsch SM, Thacker SB. Planning a public health surveillance system. Epidemiological Bulletin: Pan American Health Organization 1995;16:1–6.
8. Thacker SB, Stroup DF. Future directions for comprehensive public health surveillance and health information systems in the United States. Am J Epidemiol 1994;140:383–97.
9. Yasnoff WA, O'Carroll PW, Koo D, Linkins RW, Kilbourne EM. Public health informatics: improving and transforming public health in the information age. J Public Health Management Practice 2000;6:63–71.
10. CDC. An ounce of prevention: what are the returns? 2nd ed. Atlanta, GA: Department of Health and Human Services, CDC, 1999.
11. CDC. Impact of vaccines universally recommended for children—United States, 1990–1998. MMWR 1999;48:243–8.
12. Hinman AR, Koplan JP. Pertussis and pertussis vaccine: reanalysis of benefits, risks, and costs. JAMA 1984;251:3109–13.
13. Institute of Medicine, Committee on Summary Measures of Population Health. In: Field MJ, Gold MR, eds. Summarizing population health: directions for the development and application of population metrics. Washington, DC: National Academy Press, 1998. Available at <http://books.nap.edu/index.html. Accessed February 2001.
14. Dean AG, West DJ, Weir WM. Measuring loss of life, health, and income due to disease and injury. Public Health Rep 1982;97:38–47.
15. Vilnius D, Dandoy S. A priority rating system for public health programs. Public Health Rep 1990;105:463–70.
16. CDC. Case definitions for infectious conditions under public health surveillance. MMWR 1997;46(RR-10).
17. Council of State and Territorial Epidemiologists. Position statements. Available at <http://www.cste.org/position_statements.htm >. Accessed May 7, 2001.
18. Morris G, Snider D, Katz M. Integrating public health information and surveillance systems. J Public Health Management Practice 1996;2:24–7.
19. Meriwether RA. Blueprint for a National Public Health Surveillance System for the 21st century. J Public Health Management Practice 1996;2(4):16–23.
20. Zachman JA. A framework for information systems architecture. IBM Systems J 1987;26(3).
21. Sowa JF, Zachman JA. Extending and formalizing the framework for information systems architecture. IBM Systems J 1992;31(3).
22. Health Information and Surveillance System Board. Public Health Conceptual Data Model (PHCDM). Available at <http://www.cdc.gov/od/hissb/docs/phcdm.htm >. Accessed May 7, 2001.
23. Koo D, Parrish RG II. The changing health-care information infrastructure in the United States: opportunities for a new approach to public health surveillance. In: Teutsch SM, Churchill RE, eds. Principles and practice of public health surveillance, 2nd ed. New York, NY: Oxford University Press, 2000.
24. Data Interchange Standards Association. X12 Standards, release 4020. Alexandria, VA: Accredited Standards Committee X12, 1998. Available at <http://www.disa.org>. Accessed May 7, 2001.
25. Health Level Seven. Available at <http://www.hl7.org >. Accessed May 7, 2001.
26. Health Care Financing Administration. The Health Insurance Portability and Accountability Act of 1996 (HIPAA). Available at <http://www.hcfa.gov/hipaa/hipaahm.htm>. Accessed May 7, 2001.

27. Humphreys BL, Lindberg DAB, Schoolman HM, Barnett GO. The unified medical language system: an informatics research collaboration. JAMIA 1998;5:1–11.
28. College of American Pathologists. Systematized nomenclature of human and veterinary medicine (SNOMED®), version 3.5. Northfield, IL: College of American Pathologists. Available at <http://www.snomed.org/snomed35_txt.html>. Accessed May 7, 2001.
29. Koo D, Wetterhall SF. History and current status of the National Notifiable Diseases Surveillance System. J Public Health Management Practice 1996;2:4–10.
30. Council of State and Territorial Epidemiologists. Data release guidelines of the Council of State and Territorial Epidemiologists for the National Public Health Surveillance System. Atlanta, GA: Council of State and Territorial Epidemiologists, June 1996. Available at <http://www.cste.org/>. Accessed February 2001.
31. Privacy Law Advisory Committee, Model State Public Health Privacy Project. Model state public health privacy act. Washington, DC: Georgetown University Law Center, 1999. Available at <http://www.critpath.org/msphpa/privacy.htm>. Accessed May 7, 2001.
32. Federal Committee on Statistical Methodology, Subcommittee on Disclosure Limitation Methodology. Statistical Policy Working Paper 22: report on statistical disclosure limitation methodology. Washington, DC: Office of Management and Budget, May 1994 (PB94-165305). Available at <http://www.ntis.gov/>. Accessed May 7, 2001.
33. Vogt RL, LaRue D, Klaucke DN, Jillson DA. Comparison of an active and passive surveillance system of primary care providers for hepatitis, measles, rubella, and salmonellosis in Vermont. Am J Public Health 1983;73:795–7.
34. Hinds MW, Skaggs JW, Bergeisen GH. Benefit-cost analysis of active surveillance of primary care physicians for hepatitis A. Am J Public Health 1985;75:176–7.
35. Morris S, Gray A, Noone A, Wiseman M, Jathanna S. The costs and effectiveness of surveillance of communicable disease: a case study of HIV and AIDS in England and Wales. J Public Health Med 1996;18:415–22.
36. Haddix AC, Teutsch SM, Shaffer PA, Duñet, DO, eds. Prevention effectiveness: a guide to decision analysis and economic evaluation. New York, NY: Oxford University Press, 1996.
37. Department of Health and Human Services, Health Resources and Services Administration. Title V (Title V IS) information system web site. Accessed at <http://www.mchdata.net/>. Accessed May 7, 2001.
38. US Department of Health and Human Services. Healthy people 2010 (conference ed, 2 vols). Washington, DC: US Department of Health and Human Services, 2000.
39. Behavioral Risk Factor Surveillance System. Available at <http://www.cdc.gov/nccdphp/brfss/>. Accessed May 7, 2001.
40. Klevens RM, Fleming PL, Neal JJ, Mode of Transmission Validation Study Group. Is there really a heterosexual AIDS epidemic in the United States? Findings from a multisite validation study, 1992-1995. Am J Epidemiol 1999;149:75–84.
41. Fox J, Stahlsmith L, Remington P, Tymus T, Hargarten S. The Wisconsin firearm-related injury surveillance system. Am J Prev Med 1998;15:101–8.
42. Phillips-Howard PA, Mitchell J, Bradley DJ. Validation of malaria surveillance case reports: implications for studies of malaria risk. J Epidemiol Community Health 1990;44:155–61.
43. Weinstein MC, Fineberg HV. Clinical decision analysis. Philadelphia, PA: W.B. Saunders, 1980:84–94.
44. CDC. Manual for the surveillance of vaccine-preventable diseases. Atlanta, GA: Department of Health and Human Services, CDC, September 1999.
45. Bender JB, Hedberg CW, Besser JM, MacDonald KL, Osterholm MT. Surveillance for *Escherichia coli* 0157:H7 infections in Minnesota by molecular subtyping. N Engl J Med 1997;337:388–94.
46. Chandra Sekar C, Deming WE. On a method of estimating birth and death rates and the extent of registration. J Am Stat Assoc 1949;44:101–15.

47. Emori TG, Edwards JR, Culver DH, et al. Accuracy of reporting nosocomial infections in intensive-care–unit patients to the National Nosocomial Infections Surveillance System: a pilot study. Infect Control Hosp Epidemiol 1998;19:308–16.
48. Johnson RL, Gabella BA, Gerhart KA, McCray J, Menconi JC, Whiteneck GG. Evaluating sources of traumatic spinal cord injury surveillance data in Colorado. Am J Epidemiol 1997;146:266–72.
49. Watkins ML, Edmonds L, McClearn A, Mullins L, Mulinare J, Khoury M. The surveillance of birth defects: the usefulness of the revised US standard birth certificate. Am J Public Health 1996;86:731–4.
50. Payne SMC, Seage GR III, Oddleifson S, et al. Using administratively collected hospital discharge data for AIDS surveillance. Ann Epidemiol 1995;5:337–46.
51. Van Tuinen M, Crosby A. Missouri firearm-related injury surveillance system. Am J Prev Med 1998;15:67–74.
52. Hook EB, Regal RR. The value of capture-recapture methods even for apparent exhaustive surveys. Am J Epidemiol 1992;135:1060–7.
53. Gazarian M, Williams K, Elliott E, et al. Evaluation of a national surveillance unit. Arch Dis Child 1999;80:21–7.
54. Singh J, Foster SO. Sensitivity of poliomyelitis surveillance in India. Indian J Pediatr 1998;35:311–5.
55. Last JM, Abramson JH, Friedman GD, Porta M, Spasoff RA, Thuriaux M, eds. A dictionary of epidemiology, 3rd ed. New York, NY: Oxford University Press, 1995:173–4.
56. German RR. Sensitivity and predictive value positive measurements for public health surveillance systems. Epidemiology 2000;11:720–7.
57. Hedegaard H, Wake M, Hoffman R. Firearm-related injury surveillance in Colorado. Am J Prev Med 1998;15(3S):38–45.
58. Mähönen M, Salomaa V, Brommels M, et al. The validity of hospital discharge register data on coronary heart disease in Finland. Eur J Epidemiol 1997;13:403–15.
59. LeMier M, Cummings P, Keck D, Stehr-Green J, Ikeda R, Saltzman L. Washington state gunshot-wound surveillance system. Am J Prev Med 1998;15(3S):92–100.
60. Kimball AM, Thacker SB, Levy ME. *Shigella* surveillance in a large metropolitan area: assessment of a passive reporting system. Am J Public Health 1980;70:164–6.
61. Thacker SB, Redmond S, Rothenberg RB, Spitz SB, Choi K, White MC. A controlled trial of disease surveillance strategies. Am J Prev Med 1986;2:345–50.
62. McClure RJ, Burnside J. The Australian Capital Territory Injury Surveillance and Prevention Project. Acad Emerg Med 1995;2:529–34.
63. US Bureau of the Census. Federal-State Cooperative Program for Population Estimates: Operational Guidelines. Washington, DC: US Bureau of the Census, July 1992. Available at <http://www.census.gov/population/www/fscpp/fscpp.html>. Accessed February 2001.
64. Hahn RA, Stroup DF. Race and ethnicity in public health surveillance: criteria for the scientific use of social categories. Public Health Rep 1994;109:7–15.
65. CDC. New population standard for age-adjusting death rates. MMWR 1999;48:126–7.
66. Alter MJ, Mares A, Hadler SC, Maynard JE. The effect of underreporting on the apparent incidence and epidemiology of acute viral hepatitis. Am J Epidemiol 1987;125:133–9.
67. Romaguera RA, German RR, Klaucke DN. Evaluating public health surveillance. In: Teutsch SM, Churchill RE, eds. Principles and practice of public health surveillance, 2nd ed. New York, NY: Oxford University Press, 2000.
68. Rosenberg ML. *Shigella* surveillance in the United States, 1975. J Infect Dis 1977;136:458–60.
69. CDC. Preventing lead poisoning in young children: a statement by the Centers for Disease Control—October 1991. Atlanta, GA: Department of Health and Human Services, Public Health Service, CDC, 1991.

70. Maiese DR. Data challenges and successes with healthy people. Hyattsville, MD: Department of Health and Human Services, CDC, National Center for Health Statistics. Healthy People 2000 Statistics and Surveillance 1998. (no. 9).
71. Effler P, Ching-Lee M, Bogard A, Ieong M-C, Nekomoto T, Jernigan D. Statewide system of electronic notifiable disease reporting from clinical laboratories: comparing automated reporting with conventional methods. JAMA 1999;282:1845–50.
72. Yokoe DS, Subramanyan GS, Nardell E, Sharnprapai S, McCray E, Platt R. Supplementing tuberculosis surveillance with automated data from health maintenance organizations. Emerg Infect Dis 1999:5:779–87.
73. Johnson AM Jr, Malek M. Survey of software tools for evaluating reliability, availability, and serviceability. Association for Computing Machinery Surveys 1988;20(4).
74. Snider DE, Stroup DF. Ethical issues. In: Teutsch SM, Churchill RE, eds. Principles and practice of public health surveillance, 2nd ed. New York, NY: Oxford University Press, 2000.
75. Singleton JA, Lloyd JC, Mootrey GT, Salive ME, Chen RT, VAERS Working Group. An overview of the vaccine adverse event reporting system (VAERS) as a surveillance system. Vaccine 1999;17:2908–17.

Appendix A.

Checklist for Evaluating Public Health Surveillance Systems

Tasks for evaluating a surveillance system*	Page(s) in this report
☐ Task A. Engage the stakeholders in the evaluation	4
☐ Task B. Describe the surveillance system to be evaluated	4–11
☐ 1. Describe the public health importance of the health-related event under surveillance	4– 5
☐ a. Indices of frequency	
☐ b. Indices of severity	
☐ c. Disparities or inequities associated with the health-related event	
☐ d. Costs associated with the health-related event	
☐ e. Preventability	
☐ f. Potential future clinical course in the absence of an intervention	
☐ g. Public interest	
☐ 2. Describe the purpose and operation of the surveillance system	5–10
☐ a. Purpose and objectives of the system	
☐ b. Planned uses of the data from the system	
☐ c. Health-related event under surveillance, including case definition	
☐ d. Legal authority for data collection	
☐ e. The system resides where in organization(s)	
☐ f. Level of integration with other systems, if appropriate	
☐ g. Flow chart of system	
☐ h. Components of system	
☐ 1) Population under surveillance	
☐ 2) Period of time of data collection	
☐ 3) Data collection	
☐ 4) Reporting sources of data	
☐ 5) Data management	
☐ 6) Data analysis and dissemination	
☐ 7) Patient privacy, data confidentiality, and system security	
☐ 8) Records management program	
☐ 3. Describe the resources used to operate the surveillance system	10–11
☐ a. Funding source(s)	
☐ b. Personnel requirements	
☐ c. Other resources	
☐ Task C. Focus the evaluation design	11–12
☐ 1. Determine the specific purpose of the evaluation	
☐ 2. Identify stakeholders who will receive the findings and recommendations of the evaluation	
☐ 3. Consider what will be done with the information generated from the evaluation	
☐ 4. Specify the questions that will be answered by the evaluation	
☐ 5. Determine standards for assessing the performance of the system	
☐ Task D. Gather credible evidence regarding the performance of the surveillance system	13–24
☐ 1. Indicate the level of usefulness	13–14
☐ 2. Describe each system attribute	14–24
☐ a. Simplicity	
☐ b. Flexibility	
☐ c. Data quality	
☐ d. Acceptability	
☐ e. Sensitivity	
☐ f. Predictive value positive	
☐ g. Representativeness	
☐ h. Timeliness	
☐ i. Stability	
☐ Task E. Justify and state conclusions, and make recommendations	24
☐ Task F. Ensure use of evaluation findings and share lessons learned	25

* Adapted from *Framework for Program Evaluation in Public Health* [CDC. Framework for program evaluation in public health. MMWR 1999;48(RR-11)] and the original guidelines [CDC. Guidelines for evaluating surveillance systems. MMWR 1988;37(No. S-5)].

Appendix B.

Cross-reference of Tasks and Relevant Standards

Tasks for evaluating a surveillance system*	Relevant standards[†]
Task A. Engage the stakeholders in the evaluation.	***Stakeholder identification.*** Persons involved in or affected by the evaluation should be identified so that their needs can be addressed.<br>***Evaluator credibility.*** The persons conducting the evaluation should be trustworthy and competent in performing the evaluation to ensure that findings from the evaluation achieve maximum credibility and acceptance.<br>***Formal agreements.*** If applicable, all principal parties involved in an evaluation should agree in writing to their obligations (i.e., what is to be done, how, by whom, and when) so that each party must adhere to the conditions of the agreement or renegotiate them.<br>***Rights of human subjects.*** The evaluation should be designed and conducted in a manner that respects and protects the rights and welfare of human subjects.<br>***Human interactions.*** Evaluators should interact respectfully with other persons associated with an evaluation so that participants are not threatened or harmed.<br>***Conflict of interest.*** Conflict of interest should be handled openly and honestly so that the evaluation processes and results are not compromised.<br>***Metaevaluation.*** The evaluation should be formatively and summatively evaluated against these and other pertinent standards to guide its conduct appropriately and, on completion, to enable close examination of its strengths and weaknesses by stakeholders.
Task B. Describe the surveillance system to be evaluated.	***Complete and fair assessment.*** The evaluation should be complete and fair in its examination and recording of strengths and weaknesses of the system so that strengths can be enhanced and problem areas addressed.<br>***System documentation.*** The system being evaluated should be documented clearly and accurately.<br>***Context analysis.*** The context in which the system exists should be examined in enough detail to identify probable influences on the system.<br>***Metaevaluation.*** The evaluation should be formatively and summatively evaluated against these and other pertinent standards to guide its conduct appropriately and, on completion, to enable close examination of its strengths and weaknesses by stakeholders.
Task C. Focus the evaluation design.	***Evaluation impact.*** Evaluations should be planned, conducted, and reported in ways that encourage follow-through by stakeholders to increase the likelihood of the evaluation being used.

Appendix B. — Continued

Cross-reference of Tasks and Relevant Standards

Tasks for evaluating a surveillance system*	Relevant standards†
Task C. (*Continued*) Focus the evaluation design.	***Practical procedures.*** Evaluation procedures should be practical while needed information is being obtained to keep disruptions to a minimum.<br>***Political viability.*** During the planning and conducting of the evaluation, consideration should be given to the varied positions of interest groups so that their cooperation can be obtained and possible attempts by any group to curtail evaluation operations or to bias or misapply the results can be averted or counteracted.<br>***Cost-effectiveness.*** The evaluation should be efficient and produce valuable information to justify expended resources.<br>***Service orientation.*** The evaluation should be designed to assist organizations in addressing and serving effectively the needs of the targeted participants.<br>***Complete and fair assessment.*** The evaluation should be complete and fair in its examination and recording of strengths and weaknesses of the system so that strengths can be enhanced and problem areas addressed.<br>***Fiscal responsibility.*** The evaluator's allocation and expenditure of resources should reflect sound accountability procedures by being prudent and ethically responsible so that expenditures are accountable and appropriate.<br>***Described purpose and procedures.*** The purpose and procedures of the evaluation should be monitored and described in enough detail to identify and assess them. The purpose of evaluating a surveillance system is to promote the best use of public health resources by ensuring that only important problems are under surveillance and that surveillance systems operate efficiently.<br>***Metaevaluation.*** The evaluation should be formatively and summatively evaluated against these and other pertinent standards to guide its conduct appropriately and, on completion, to enable close examination of its strengths and weaknesses by stakeholders.
Task D. Gather credible evidence regarding the performance of the surveillance system.	***Information scope and selection.*** Information collected should address pertinent questions regarding the system and be responsive to the needs and interests of clients and other specified stakeholders.<br>***Defensible information sources.*** Sources of information used in the system evaluation should be described in enough detail to assess the adequacy of the information.

Appendix B. — Continued

Cross-reference of Tasks and Relevant Standards

Tasks for evaluating a surveillance system*	Relevant standards†
Task D. (*Continued*) Gather credible evidence regarding the performance of the surveillance system.	***Valid information.*** Information-gathering procedures should be developed and implemented to ensure a valid interpretation for the intended use.<br>***Reliable information.*** Information-gathering procedures should be developed and implemented to ensure sufficiently reliable information for the intended use.<br>***Systematic information.*** Information collected, processed, and reported in an evaluation should be systematically reviewed and any errors corrected.<br>***Metaevaluation.*** The evaluation should be formatively and summatively evaluated against these and other pertinent standards to guide its conduct appropriately and, on completion, to enable close examination of its strengths and weaknesses by stakeholders.
Task E. Justify and state conclusions, and make recommendations.	***Values identification.*** The perspectives, procedures, and rationale used to interpret the findings should be carefully described so that the bases for value judgments are clear.<br>***Analysis of information.*** Information should be analyzed appropriately and systematically so that evaluation questions are answered effectively.<br>***Justified conclusions.*** Conclusions that are reached should be explicitly justified for stakeholders' assessment.<br>***Metaevaluation.*** The evaluation should be formatively and summatively evaluated against these and other pertinent standards to guide its conduct appropriately and, on completion, to enable close examination of its strengths and weaknesses by stakeholders.
Task F. Ensure use of evaluation findings and share lessons learned.	***Evaluator credibility.*** The persons conducting the evaluation should be trustworthy and competent in performing the evaluation to ensure that findings from the evaluation achieve maximum credibility and acceptance.<br>***Report clarity.*** Evaluation reports should clearly describe the system being evaluated, including its context and the purposes, procedures, and findings of the evaluation so that essential information is provided and easily understood.<br>***Report timeliness and dissemination.*** Substantial interim findings and evaluation reports should be disseminated to intended users so that they can be used in a timely fashion.

Appendix B. — Continued

Cross-reference of Tasks and Relevant Standards

Tasks for evaluating a surveillance system*	Relevant standards†
Task F. Ensure use of the findings of the evaluation and share lessons learned.	***Evaluation impact.*** Evaluations should be planned, conducted, and reported in ways that encourage follow-through by stakeholders to increase the likelihood of the evaluation being used.<br>***Disclosure of findings.*** The principal parties of an evaluation should ensure that the full evaluation findings with pertinent limitations are made accessible to the persons affected by the evaluation and any others with expressed legal rights to receive the results.<br>***Impartial reporting.*** Reporting procedures should guard against the distortion caused by personal feelings and biases of any party involved in the evaluation so that the evaluation reflects the findings fairly.<br>***Metaevaluation.*** The evaluation should be formatively and summatively evaluated against these and other pertinent standards to guide its conduct appropriately and, on completion, to enable close examination of its strengths and weaknesses by stakeholders.

* Adapted from *Framework for Program Evaluation in Public Health* [CDC. Framework for program evaluation in public health. MMWR 1999;48(RR–11)] and the original guidelines [CDC. Guidelines for evaluating surveillance systems. MMWR 1988;37(No. S-5)].

† Adapted from *Framework for Program Evaluation in Public Health* [CDC. Framework for program evaluation in public health. MMWR 1999;48(RR-11)].

July 27, 2001 / Vol. 50 / No. RR-13

MORBIDITY AND MORTALITY WEEKLY REPORT

Recommendations and Reports

Continuing Education Activity Sponsored by CDC

Updated Guidelines for Evaluating Surveillance Systems Recommendations from the Guidelines Working Group

EXPIRATION — July 27, 2002

You must complete and return the response form electronically or by mail by **July 27, 2002,** to receive continuing education credit. If you answer all of the questions, you will receive an award letter for 1.75 hours Continuing Medical Education (CME) credit, .15 hour Continuing Education Units (CEUs), or 1.9 hours Continuing Nursing Education (CNE) credit. If you return the form electronically, you will receive educational credit immediately. If you mail the form, you will receive educational credit in approximately 30 days. No fees are charged for participating in this continuing education activity.

INSTRUCTIONS

By Internet

1. Read this *MMWR* (Vol. 50, RR-13), which contains the correct answers to the questions beginning on the next page.
2. Go to the *MMWR* Continuing Education Internet site at <http://www.cdc.gov/mmwr/cme/conted.html>.
3. Select which exam you want to take and select whether you want to register for CME, CEU, or CNE credit.
4. Fill out and submit the registration form.
5. Select exam questions. To receive continuing education credit, you must answer all of the questions. Questions with more than one correct answer will instruct you to "Indicate all that apply."
6. Submit your answers no later than **July 27, 2002**.
7. Immediately print your Certificate of Completion for your records.

By Mail or Fax

1. Read this *MMWR* (Vol. 50, RR-13), which contains the correct answers to the questions beginning on the next page.
2. Complete all registration information on the response form, including your name, mailing address, phone number, and e-mail address, if available.
3. Indicate whether you are registering for CME, CEU, or CNE credit.
4. Select your answers to the questions, and mark the corresponding letters on the response form. To receive continuing education credit, you must answer all of the questions. Questions with more than one correct answer will instruct you to "Indicate all that apply."
5. Sign and date the response form or a photocopy of the form and send no later than **July 27, 2002**, to
   Fax: 404-639-4198 Mail: MMWR CE Credit
   Office of Scientific and Health Communications
   Epidemiology Program Office, MS C-08
   Centers for Disease Control and Prevention
   1600 Clifton Rd, N.E.
   Atlanta, GA 30333
6. Your Certificate of Completion will be mailed to you within 30 days.

ACCREDITATION

Continuing Medical Education (CME). CDC is accredited by the Accreditation Council for Continuing Medical Education (ACCME) to provide continuing medical education for physicians. CDC designates this educational activity for a maximum of 1.75 hours in category 1 credit toward the AMA Physician's Recognition Award. Each physician should claim only those hours of credit that he/she actually spent in the educational activity.

Continuing Education Unit (CEU). CDC has been approved as an authorized provider of continuing education and training programs by the International Association for Continuing Education and Training and awards .15 hour Continuing Education Units (CEUs).

Continuing Nursing Education (CNE). This activity for 1.9 contact hours is provided by CDC, which is accredited as a provider of continuing education in nursing by the American Nurses Credentialing Center's Commission on Accreditation.

GOAL AND OBJECTIVES

This MMWR updates methods and tasks for evaluating a public health surveillance system. The goal of this report is to guide public health personnel and other interested parties through the assessment of a surveillance system using the *Framework for Program Evaluation in Public Health* in addition to the elements in the original guidelines for surveillance evaluation published in 1988. Upon completion of this educational activity, the reader should be able to a) outline the six tasks necessary to evaluate a surveillance system; b) list and define the nine system attributes that provide credible evidence of a system's performance; and c) describe steps for making recommendations and ensuring use of the evaluation's findings.

To receive continuing education credit, please answer all of the following questions.

1. Data from a public health surveillance system can be used to

A. Monitor trends in the burden of disease, including detection of epidemics.
B. Measure the burden of disease, including changes in populations at risk.
C. Prioritize allocation of health resources.
D. Provide the basis for epidemiologic research.
E. All of the above.

2. Because of advances in technology and information architecture, new concerns for evaluation of surveillance systems include

A. User interface.
B. Adherence to confidentiality and security standards.
C. Data format and coding.
D. All of the above.
E. A and C.

3. Engaging stakeholders in the evaluation process will help ensure all of the following except

A. That the evaluation will address appropriate questions.
B. That pertinent attributes will be described and addressed.
C. That the findings will be acceptable and useful.
D. That the effect of the recommended changes will be minimal.
E. That persons or organizations that have an investment in the system will be represented.

4. A case definition for the health-related event under surveillance can include all of the following except

A. Level of diagnostic certainty.
B. Level of statistical certainty.
C. Epidemiologic information.
D. Clinical manifestations of the health-related event.
E. Laboratory results.

5. Gathering credible evidence regarding the system's performance might include which of the following?

A. Data quality.
B. Acceptability.
C. Sensitivity.
D. Stability.
E. All of the above.

6. Sensitivity of a surveillance system describes

A. The ability of the system to detect outbreaks.
B. The likelihood that a case reported to the surveillance system is a true case.
C. The proportion of cases of a health event detected by the surveillance system.
D. All of the above.
E. A and C.

7. Representativeness of a surveillance system

A. Is not affected by case ascertainment bias.
B. Can be assessed by comparing characteristics of reported events to all such events.
C. Does not affect the generalizability of the surveillance system.
D. All of the above.
E. A and C.

8. Conclusions of and recommendations from the evaluation of the surveillance system

A. Should be made based on credible evidence collected throughout the evaluation.
B. Should state whether the system is meeting its objectives.
C. Should not recommend discontinuation of a system.
D. All of the above.
E. A and B.

9. Ensuring the use of the evaluation's findings is enhanced by

A. Deliberate effort to disseminate the findings.
B. Excluding stakeholders from development of recommendations.
C. Reporting findings in a single and consistent format.
D. Disseminating findings only to those who can carry out recommendations.
E. Including a diagram that describes the data flow within the system.

10. Indicate your work setting.

A. State/local health department.
B. Other public health setting.
C. Hospital clinic/private practice.
D. Managed care organization.
E. Academic institution.
F. Other.

11. Which best describes your professional activities?

A. Patient care — emergency/urgent care department.
B. Patient care — inpatient.
C. Patient care — primary-care clinic or office.
D. Laboratory/pharmacy.
E. Public health.
F. Other.

12. I plan to use these recommendations as the basis for ... *(Indicate all that apply.)*

A. health education materials.
B. insurance reimbursement policies.
C. local practice guidelines.
D. public policy.
E. other.

13. Each year, approximately how many public health surveillance systems do you evaluate?

A. None.
B. 1
C. 2
D. 3
E. 4
F. $\geq$5

14. How much time did you spend reading this report and completing the exam?

A. 1–1.5 hours.
B. More than 1.5 hours but fewer than 2 hours.
C. 2–2.5 hours.
D. More than 2.5 hours.

15. After reading this report, I am confident that I can outline the six tasks necessary to evaluate a surveillance system.

A. Strongly agree.
B. Agree.
C. Neither agree nor disagree.
D. Disagree.
E. Strongly disagree.

16. After reading this report, I am confident that I can list and define the nine system attributes that provide credible evidence of a system's performance.

A. Strongly agree.
B. Agree.
C. Neither agree nor disagree.
D. Disagree.
E. Strongly disagree.

17. After reading this report, I am confident that I can describe steps for making recommendations and ensuring use of the evaluation's findings.

A. Strongly agree.
B. Agree.
C. Neither agree nor disagree.
D. Disagree.
E. Strongly disagree.

18. The objectives are relevant to the goal of this report.

A. Strongly agree.
B. Agree.
C. Neither agree nor disagree.
D. Disagree.
E. Strongly disagree.

19. The tables, figures, and appendices are useful.

A. Strongly agree.
B. Agree.
C. Neither agree nor disagree.
D. Disagree.
E. Strongly disagree.

20. Overall, the presentation of the report enhances my ability to understand the material.

A. Strongly agree.
B. Agree.
C. Neither agree nor disagree.
D. Disagree.
E. Strongly disagree.

21. These recommendations will affect my practice.

A. Strongly agree.
B. Agree.
C. Neither agree nor disagree.
D. Disagree.
E. Strongly disagree.

22. How did you learn about this continuing education activity?

A. Internet.
B. Advertisement (e.g., fact sheet, *MMWR* cover, newsletter, or journal).
C. Coworker/supervisor.
D. Conference presentation.
E. *MMWR* subscription.
F. Other.

Correct answers for questions 1–9

1.E 2.D 3.D 4.B 5.E 6.E 7.B 8.E 9.A

MMWR Response Form for Continuing Education Credit July 27, 2001/Vol. 50/No. RR-13

Updated Guidelines for Evaluating Public Health Surveillance Systems Recommendations from the Guidelines Working Group

To receive continuing education credit, you must
1. provide your contact information;
2. indicate your choice of CME, CEU, or CNE credit;
3. answer all of the test questions;
4. sign and date this form or a photocopy;
5. submit your answer form by July 27, 2002.
Failure to complete these items can result in a delay or rejection of your application for continuing education credit.

Detach or photocopy.

Last Name *First Name*

Street Address or P.O. Box

Apartment or Suite

City *State* *ZIP Code*

Phone Number *Fax Number*

E-Mail Address

Check One

- [ ] *CME Credit*
- [ ] *CEU Credit*
- [ ] *CNE Credit*

Fill in the appropriate blocks to indicate your answers. Remember, you must answer all of the questions to receive continuing education credit!

1. [ ] A [ ] B [ ] C [ ] D [ ] E	12. [ ] A [ ] B [ ] C [ ] D [ ] E
2. [ ] A [ ] B [ ] C [ ] D [ ] E	13. [ ] A [ ] B [ ] C [ ] D [ ] E [ ] F
3. [ ] A [ ] B [ ] C [ ] D [ ] E	14. [ ] A [ ] B [ ] C [ ] D
4. [ ] A [ ] B [ ] C [ ] D [ ] E	15. [ ] A [ ] B [ ] C [ ] D [ ] E
5. [ ] A [ ] B [ ] C [ ] D [ ] E	16. [ ] A [ ] B [ ] C [ ] D [ ] E
6. [ ] A [ ] B [ ] C [ ] D [ ] E	17. [ ] A [ ] B [ ] C [ ] D [ ] E
7. [ ] A [ ] B [ ] C [ ] D [ ] E	18. [ ] A [ ] B [ ] C [ ] D [ ] E
8. [ ] A [ ] B [ ] C [ ] D [ ] E	19. [ ] A [ ] B [ ] C [ ] D [ ] E
9. [ ] A [ ] B [ ] C [ ] D [ ] E	20. [ ] A [ ] B [ ] C [ ] D [ ] E
10. [ ] A [ ] B [ ] C [ ] D [ ] E [ ] F	21. [ ] A [ ] B [ ] C [ ] D [ ] E
11. [ ] A [ ] B [ ] C [ ] D [ ] E [ ] F	22. [ ] A [ ] B [ ] C [ ] D [ ] E [ ] F

Signature ***Date I Completed Exam***

Use of trade names and commercial sources is for identification only and does not imply endorsement by the U.S. Department of Health and Human Services.

References to non-CDC sites on the Internet are provided as a service to *MMWR* readers and do not constitute or imply endorsement of these organizations or their programs by CDC or the U.S. Department of Health and Human Services. CDC is not responsible for the content of pages found at these sites.

The *Morbidity and Mortality Weekly Report (MMWR)* Series is prepared by the Centers for Disease Control and Prevention (CDC) and is available free of charge in electronic format and on a paid subscription basis for paper copy. To receive an electronic copy on Friday of each week, send an e-mail message to *listserv@listserv.cdc.gov*. The body content should read *SUBscribe mmwr-toc*. Electronic copy also is available from CDC's World-Wide Web server at *http://www.cdc.gov/mmwr/* or from CDC's file transfer protocol server at *ftp://ftp.cdc.gov/pub/Publications/mmwr/*. To subscribe for paper copy, contact Superintendent of Documents, U.S. Government Printing Office, Washington, DC 20402; telephone (202) 512-1800.

Data in the weekly *MMWR* are provisional, based on weekly reports to CDC by state health departments. The reporting week concludes at close of business on Friday; compiled data on a national basis are officially released to the public on the following Friday. Address inquiries about the *MMWR* Series, including material to be considered for publication, to: Editor, *MMWR* Series, Mailstop C-08, CDC, 1600 Clifton Rd., N.E., Atlanta, GA 30333; telephone (888) 232-3228.

All material in the *MMWR* Series is in the public domain and may be used and reprinted without permission; citation as to source, however, is appreciated.

☆U.S. Government Printing Office: 2001-633-173/48243 Region IV
