# Supplementary material for: Evaluation of the influenza-like illness sentinel surveillance system: A national perspective in Tanzania from January to December 2019
Source: PLoS One. 2023 Mar 20;18(3):e0283043. doi: 10.1371/journal.pone.0283043 (PMC10027206; doi:10.1371/journal.pone.0283043)
Supplement: S3 File — (PDF) [file pone.0283043.s004.pdf]

Verification of number of samples sent to National Public Health Laboratory (NPHL) by using registers from sentinel site books and Laboratory information system (LIS), 2019.

Month (in 2019)	SBNH		DRRH		MRRH	
	**S1**	**S2**	**S3**	**S4**	**S5**	**S6**
January	51	35	7	5	51	48
February	22	54	18	8	78	62
March	26	14	2	15	47	59
April	21	23	6	7	82	30
May	12	24	0	0	41	111
June	10	0	13	0	20	0
July	1	0	10	21	71	18
August	4	0	8	0	16	65
September	4	0	6	0	63	0
October	18	0	9	0	24	4
November	24	0	0	0	46	0
December	3	10	0	3	47	50
Total	196	160	79	56	586	447
Standard dev.	15.70		6.3		29.0	
% Consistency	81.60% (160/196)		70.90% (56/79)		76.30% (447/586)	

Overall % consistency =77% (663/861) – [target; ≥95%]
Overall % discrepancy=23% (198/861) – [target; <5%]

Key: **S1**: Number of samples sent from St Benedict's Ndanda Hospital (SBNH) to National Public Health Laboratory (NPHL) by using SBNH sentinel site book, **S2**: Number of samples sent from SBNH to NPHL by using NPHL sample receiving Laboratory Information System (LIS), **S3**: Number of samples sent from Dodoma Regional Referral Hospital (DRRH) to NPHL by using DRRH sentinel site book, **S4**: Number of samples sent from DRRH to NPHL by using NPHL sample receiving LIS, **S5**: Number of samples sent from Mwananyamala Regional Referral Hospital (MRRH) to NPHL by using MRRH sentinel site book, **S6**: Number of samples sent from MRRH to NPHL by using NPHL sample receiving LIS.
