# Supplementary material for: Evaluation of the influenza-like illness sentinel surveillance system: A national perspective in Tanzania from January to December 2019
Source: PLoS One. 2023 Mar 20;18(3):e0283043. doi: 10.1371/journal.pone.0283043 (PMC10027206; doi:10.1371/journal.pone.0283043)
Supplement: Attachment — Submitted filename: PONE-D-22-16315_review.docx [file pone.0283043.s006.docx]

PLOS ONE

Evaluation of the influenza-like illness sentinel surveillance system: A national perspective in Tanzania from January to December 2019

The manuscript **Evaluation of the influenza-like illness sentinel surveillance system: A national perspective in Tanzania from January to December 2019 has been reviewed.**

**Fu¡ind below corrections and commentaris :**

**Abstract**

# Background

estimating the distribution of the disease and identifying any circulating viral strains with pandemic potential.

# Methodology

We collected retrospective data through a review of the Tanzania National Influenza Surveillance System electronic forms for 2019 and interviewed the surveillance personnel on the system’s description and operating procedure. from March to April 2021. Case definition (ILIInfluenza Like Illness and SARI-Severe Acute Respiratory Illness), results, and demographic characteristics of each patient were obtained from Laboratory Information System (Disa Lab) at National Influenza Center (NIC) of Tanzania?. The system’s attributes were evaluated using the U.S Centers for disease control and prevention (CDC) updated guidelines for evaluating public health surveillance systems (2007). Additionally, Surveillance system attributes were examined on system’s performance indicators, each being scored on a scale of 1 to 5 (very poor to very good performance).

# Results

A review of the Influenza Laboratory electronic forms from the Laboratory information system (Disa Lab) showed that a total of 1731 of both nasopharyngeal and oropharyngeal samples were collected from each influenza suspected case in 2019, from fourteen (14/14) sentinel sites of the influenza surveillance system in Tanzania.

Laboratory confirmed cases were 21.5% (373/1731) with a predictive value positive (PVP) of 21.5%. Influenza A was detected in most patients (76.1%); and cases who had ILI symptoms were 1.2 times more likely to be positive to influenza virus than those presenting with SARI [aOR=1.36 (95%CI: 1.03-1.79); p=0.031]. Thirty-seven percent of clients’ results, met the required turnaround time (TAT), and 40% of case based forms were incompletely filled. Although the accuracy of data was good (100%), data consistency was below (77%) the established target of ≥95%.

# Conclusion

The overall system performance was satisfactory in conforming with its objectives and generates accurate data with an average performance of 100%. The complexity of the system contributed to the reduced consistency of data from sentinel sites to the NPHL. Improvement of the use of the available data could be made to inform and promote preventive measures, especially among the most vulnerable population. Increasing sentinel sites would increase population coverage and level of system representativeness.

# Introduction

Influenza refers to an acute viral respiratory tract disease characterized by fever, headache, myalgia, prostration, coryza, sore throat, and cough. Cough is often severe and prolonged with recovery in 2-7 days[1].The World Health Organization (WHO) estimates that seasonal influenza causes; 3 to 5 million cases of severe influenza disease and may result in 290 000-650 000 deaths each year due to respiratory diseases alone[4]. In Tanzania, the National Influenza Sentinel Surveillance System (NISSS) was initiated in 2008 whereby on July 4, 2009 the first case was reported[2], since then, epidemics have been persisting. Currently, the documented prevalence of seasonal influenza in Tanzania is 8.0%, according to the first surveillance evaluation done 30 months after the initiation of the surveillance system[5].

Global influenza surveillance, coordinated by WHO under the Global Influenza Surveillance and Response Network (GISRN), is key to monitoring global trends of seasonal influenza virus circulation, guiding strain selection for annual influenza vaccine composition, monitoring the acquisition of resistance to antiviral drugs, detecting the emergence of influenza viruses with pandemic potential, and monitoring the spread and impact of pandemic influenza viruses[3].

The global influenza surveillance network (GISN) was initiated in 1952 by the WHO as a response strategy against influenza disease[2]. WHO recommends the use of standard case definitions and procedures for global influenza surveillance among outpatients and inpatients as well as periodic comprehensive evaluations of established surveillance systems, beginning 1–2 years after implementation[2].

Influenza surveillance system operates through an organizational structure that involves the sentinel sites, National Public Health Laboratory (NPHL), the epidemiology section of the Ministry of Health (MoH) and surveillance collaborative partners (WHO and Centers for disease control and prevention (CDC) (S1Fig).

Sentinel sites are hospitals or health centres and each sentinel site has a surveillance focal person who coordinates the functioning of the surveillance activity, linking and reporting to NPHL and Epidemiology section at the MoH. The main functions of a sentinel site are to enroll patients for surveillance, collect epidemiological and laboratory data, perform data entry and simple analysis and submit collected Case-Based Forms (CBF) and laboratory specimens to the Epidemiology section of the MoH and NPHL, respectively[2]. NPHL, as a National Influenza Center (NIC) of Tanzania, has the role to support laboratory capacity for the surveillance system at central level (MoH) and sentinel sites[2]

The Epidemiology Section of the MoH is responsible for the overall coordination, staff training, response, procurement of equipment & consumables, monitoring and evaluation and for providing feedback to the staff at all levels. They also have the primary responsibility for data management, analysis, and dissemination of reports to stakeholders[2].

Overall responsibilities of collaborative partners are to contribute to technical assistance and provide resource support for running surveillance activities [2]. The main purpose for the implementation of an influenza sentinel surveillance system includes: determining the characteristics of circulating influenza virus’ as well as detecting and ascertain new strains with pandemic potential[2].

Although influenza sentinel surveillance has been established in several African countries, data about the performance of established surveillance systems are limited on the continent and especially in Tanzania[6–8]. Such evaluations enable countries to assess the performance of their surveillance systems, identify areas for improvement and provide evidence of data reliability for policymaking and public health interventions as well as compliance with international surveillance standards.

This study aimed at evaluating the NISSS in Tanzania by determining whether the NISSS complies with its objectives stated in the protocol for national influenza sentinel surveillance system [2]. Evaluating the system’s attributes and usefulness as well as providing relevant recommendations for improvement. Findings from this evaluation will help to improve the performance of the influenza surveillance system in Tanzania.

# Materials and Methods

We described the attributes and usefulness of the National Influenza Sentinel Surveillance System (NISSS) of Tanzania using a descriptive cross-sectional survey. We extracted and evaluated routinely recorded ILI and SARI data between January and December 2019.

# Data collection procedure

Data on ILI and SARI from all fourteen sentinel facilities in Tanzania were extracted and abstracted from the NIC database (Disa Lab) into Microsoft Office Excel format. Additionally, we selected three sentinel sites, based on their location (southern, eastern and central part of the country), volume of clients enrolled and different levels of care they provided (primary to tertiary hospital), and obtained permission for site visits. These sentinel sites; Dodoma Regional Referral Hospital (DRRH), Mwananyamala Regional Referral Hospital (MRRH) and St. Benedict’s Ndanda Hospital (SBRRH), were visited for at least one week each and their records were collected and reviewed.

The researchers interviewed all personnel directly involved in National Influenza Sentinel surveillance and also partook in surveillance activities in each of the selected sentinel sites while observing practices. The staff members were interviewed using a structured questionnaire and guided interviews on their knowledge on surveillance activities (see S1 File). Tally method was used to capture data on completeness of case based forms and evaluation of surveillance system’s attributes was done using CDC guidelines for evaluating public health surveillance systems (see S2 File). The unique serial numbers on case investigation forms for each sentinel site were used to retrieve the randomized forms for examination. In addition, we collected and reviewed

surveillance registers at the sentinel sites visited.

# Population under surveillance

The NISSS cover the entire population of Tanzania, which was approximated to be 56.32 million by 2019 [9].Case management data was captured from fourteen sentinel facilities which are situated in the regions of Kigoma, Dodoma, Mwanza, Manyara, Arusha, Mtwara, Dar es Salaam and Zanzibar (see Fig 1).

# Inclusion criteria

The data of all influenza suspects detected by either Influenza-like illness (ILI) or Severe acute respiratory infections (SARI) case definition enrolled in the surveillance system and therefore included in 2019 influenza data set (electronic forms) were used for analysis. *ILI case* was defined as “An acute respiratory infection with: measured fever of ≥ 38 C° and cough; with onset within the last 10 days”. *SARI case* was defined as “An acute respiratory infection with history of fever or measured fever of ≥ 38 C°; and cough; with onset within the last 10 days; and requires hospitalization”.[2]

# Exclusion criteria

All clients whose data were missing important information such as age, sex, case definition type, name of sentinel site and influenza results were excluded from the analysis. Also stakeholders who were not willing to participate in interview were not included in this study.

# Study area

The evaluation was conducted at the MoH epidemiology section in Dodoma, NPHL in

Dar Es salaam, and three selected sentinel sites which are: SBNH in Mtwara region, DRRH in Dodoma region and MRRH in Dar es salaam region (see Fig 1). The sites were selected purposely due to location, different levels of care and relatively higher number of clients enrolled in the NISSS in Tanzania compared to other sentinel sites.

***Fig 1. Sentinel sites of the National Influenza Sentinel Surveillance System in Tanzania, 2019 (N=16).***

# Data processing and analysis

Data were entered and stored in Microsoft (MS) Excel ^®^ version 2019 and analysis was done using Microsoft Excel^®^ as well as Stata^®^ version 15.1. Logistic regression model was used to identify the risk factors for influenza positivity among the clients enrolled in the surveillance system. The variables with p value <0.05 in multivariate analysis was considered as significant predictors of influenza. Additionally, surveillance system attributes’ performance scores were described in a scale from 1 to 5 from very poor performance to very good performance respectively. The description of these scores were based on the consensus opinions of surveillance experts: virologist public health specialist and epidemiologists of the NISSS.

# Ethics statement

The evaluation was done within the framework of Integrated Disease Surveillance and Response matrix implemented by the Tanzania Ministry of Health and therefore did not have to receive formal review by Ethical Review Committees. The Field

Epidemiology and Laboratory Training Programme and National Influenza Center in Tanzania approved the study. Permission was sought and obtained from the Ministry of Health of Tanzania for the epidemiology section and authorities in the sentinel facilities before commencement of the evaluation. Data were anonymized before being accessed and all respondents provided informed, written consent and were assured of confidentiality.

**Results**

# Characteristics of the study participants

The Influenza Laboratory electronic forms from the Laboratory information system (Disa Lab) at NIC showed that; a total of 1731 both nasopharyngeal and oropharyngeal swabs specimens from suspected cases of influenza were collected from January to December, 2019 of which 912 (52.7%) were from male participants. The individuals enrolled in the surveillance system had the median age of 3 years (Interquartile range=28). Forty-one percent (708/1731) patients were enrolled in the surveillance system using SARI case definition. Forty-seven percent (83/175) of clients who presented with ILI symptoms were confirmed positive to influenza B virus, and only 5.5% (6/109) of these SARI during the time of enrolment. (see Table 1 and Fig 2). Of all samples from ILI cases, 21.5% (373/1731) were laboratory confirmed influenza cases . Most samples were collected from Mwananyamala regional referral hospital sentinel site [25.8% (447/1731)] and the least number of samples from Bombo regional hospital [0.1% (2/1731)]. Most laboratory results of people under surveillance [63.4% (1098/1731)] did not meet the turnaround time (TAT) of ≤7 days, (see Table 1). ***Fig 2.Distribution of influenza types in Tanzania according to case definition used among confirmed cases of influenza from January to December 2019 (N=373).***

**Table 1. Social demographic characteristics of the people under National Influenza** 246 **Sentinel Surveillance System in Tanzania, 2019.**

**CASE DEFINITION TYPE**

# Characteristic

# ILI n =1023 SARI (n=708) Total (N=1731)

**n (%)	n(%)	n(%)**

| **Age (years)** |  |  |  |
| --- | --- | --- | --- |
| 0-4 | 384 (37.54) | 569 (80.37) | 953 (55.05) |
| 5-14 | 101 (9.87) | 27 (3.81) | 128 (7.39) |
| 15-24 | 137 (13.39) | 23 (3.25) | 160 (9.24) |
| 25-34 | 174 (17.01) | 18 (2.54) | 192 (11.09) |
| 35-44 | 98 (9.58) | 18 (2.54) | 116 (6.70) |
| 45-54 | 47 (4.59) | 14 (1.98) | 61 (3.52) |
| 55-64 | 53 (5.18) | 15 (2.12) | 68 (3.93) |
| ≥65 | 29 (2.83) | 24 (3.39) | 53 (3.06) |
| Median  (IQR, (p75, p25) | 18  (31, 33.2) | 1  (3, 3.0) | 3  (28, 28.0) |
| **Sex** |  |  |  |
| Female | 531 (51.91) | 276 (38.98) | 807 (46.62) |
| Male | 481 (47.02) | 431 (60.88) | 912 (52.69) |
| Missing | 11 (1.08) | 1 (0.14) | 12 (0.69) |
|  |  |  |  |
| **Sentinel site** |  |  |  |
| Bombo regional hospital | 2 (0.20) | 0 (0.00) | 2 (0.12) |
| Bububu Military hospital | 184 (17.99) | 0 (0.00) | 184 (10.63) |
| Dodoma regional referral hospital | 6 (0.59) | 50 (7.06) | 56 (3.24) |
| Hydom Lutheran hospital | 47 (4.59) | 38 (5.37) | 85 (4.91) |
| International School of Tanganyika Clinic | 39 (3.81) | 0 (0.00) | 39 (2.25) |
| Istiqaama hospital | 5 (0.49) | 0 (0.00) | 5 (0.29) |
| Kibondo district hospital | 24 (2.35) | 100 (14.12) | 124 (7.16) |
| Mbalizi Military hospital | 193 (18.87) | 0 (0.00) | 193 (11.15) |
| Meru district hospital | 97 (9.48) | 41 (5.79) | 138 (7.97) |
| Mt. Meru regional referral hospital | 64 (6.26) | 99 (13.98) | 163 (9.42) |
| Mwananyamala regional referral hospital | 170 (16.62) | 277 (39.12) | 447 (25.82) |
| Mzinga hospital | 65 (6.35) | 0 (0.00) | 65 (3.76) |
| Sekou-Touré regional referral hospital | 43 (4.20) | 27 (3.81) | 70 (4.04) |
| St. Benedict’s Ndanda hospital | 84 (8.21) | 76 (10.73) | 160 (9.24) |
|  |  |  |  |
| **Influenza virus results** |  |  |  |
| Influenza A Detected | 175 (17.11) | 109 (15.40) | 284 (16.41) |
| A(H3N2) | 31 (3.03) | 23 (3.25) | 54 (3.12) |
| A(Unclassified) | 144 (14.08) | 86 (12.15) | 230 (13.29) |
| Influenza B Detected | 83 (8.11) | 6 (0.85) | 89 (5.14) |
| Influenza A and B Not Detected | 757 (74.00) | 591 (83.47) | 1348 (77.87) |
| Non-human sample (Failed results) | 8 (80.00) | 2 (0.28) | 10 (0.58) |
|  |  |  |  |
| **Turnaround time (TAT)** |  |  |  |

≤ 7 days	302 (29.52)

331 (46.75)

633 (36.57)

377 (53.25)

1098 (63.43)

>7 days	721 (70.48)

## Usefulness (Utility) of influenza surveillance system

The usefulness of the Influenza surveillance system was assessed among the stakeholders from all selected sentinel sites, and 80% (10/12) agreed on the importance of NISSS in Tanzania. Furthermore, the surveillance system provided the useful data to all stakeholders via periodic reports, including monthly and annual influenza surveillance bulletin. Additionally, national surveillance data allowed the calculation of the burden of disease and show the weekly distribution of influenza cases in the country (see Fig 3). The mean score for utility was 5.

***Fig 3.Weekly distribution of influenza cases in Tanzania from January to December 2019*** ***(N=373).***

## Simplicity

The simplicity of public health surveillance system refers to ease of operation of the system. Although all stakeholders [100% (12/12)] agreed that system was simple. However, in assessing this attribute the influenza annual reports were reviewed from all selected sentinel sites. Only 21.4% (600/2803) of all patients admitted to sentinel sites (SBNH, DRRH and MRRH) enrolled by SARI standard case definition (see Table 2).

We also observed the use of manual registers at the sentinel sites, which may influence the delay of surveillance activities and compromise the data accuracy. These data from selected sites show that the surveillance system is not simple in its operation due to its method of collecting the data. The mean score for simplicity was 3.

**Table 2. Number of SARI patients admitted and collected samples in selected** 270 **sentinel sites.**

**Sentinel site**

|  | SBNH | DRRH | MRRH | Total |
| --- | --- | --- | --- | --- |
| **P1** | 1186 | 79 | 1538 | 2803 |
| **ST** | 122 (10%) | 79 (100%) | 399(26%) | 600 (21%) |
| **AC** | 10 (13%) | 7 (9%) | 33 (41%) | 50 (63%) |

Key**: P1:** Admitted patients who fulfilled influenza case definition for SARI; **ST**: Total number of samples from SARI admitted cases from January to December 2019; **AC**: Average target set

for sample collection from January to December 2019 per month (80 samples per month), **SBNH**: St. Benedict’s Ndanda hospital; **DRRH**: Dodoma regional referral hospital; **MRRH**: Mwananyamala regional referral hospital

# Data quality

Data quality entails the completeness, accuracy, consistency and comprehensiveness of data contained in the surveillance system. In this attribute the data completeness, accuracy and data consistency examined are as follow:

## Data consistency

In evaluating this attribute; comparison was done between the number of samples found in sentinel site sample registration books with NPHL influenza registration via Laboratory information system (LIS) from January to December, 2019. The overall inconsistency (23%) of data was observed from sentinel sites to NPHL, and only 77% (663/861) of data were the same between selected sentinel sites and NPHL. The highest discrepancy was observed in samples sent from MRRH to NPHL with a standard deviation of 29.0 (see Table 3). The mean score for data consistency was 4.

## Data completeness

Data completeness target was 90% where numerator was the number of data collection forms with complete data for all variables while denominator was number of all data collection forms submitted. We observed that 60% (164/415) of the reviewed forms were filled and the remaining 40% of the forms were not filled properly, whereby most of the blanks were observed in case definition (15%) as well as date of sample collection (11%) fields, (see Fig 4). The mean score for data completeness was 3.

***Fig 4. The proportion of incompleteness of selected parameters on influenza case-based forms used by National Influenza Sentinel Surveillance System in Tanzania, 2019 (N=415).***

## Data accuracy

Through document review, we observed that; Internal Quality Control (IQC) was always done in each run of the samples and passed, also External Quality Assessment

(EQA) was performed with 100% concordance with WHO standards. Furthermore, NPHL had an average performance of 100% in all EQA panels. This entails the accurately estimation of the disease burden in the country. The mean score for data accuracy was 5.

**Table 3. Verification of number of samples sent to National Public Health** 313	**Laboratory (NPHL) by using registers from sentinel site books and Laboratory** 314	**information system (LIS), 2019.**

| **Month (in 2019)** | **SBNH** |  | **DRRH** |  | **MRRH** |  |
| --- | --- | --- | --- | --- | --- | --- |
|  | **S1** | **S2** | **S3** | **S4** | **S5** | **S6** |
| January | 51 | 35 | 7 | 5 | 51 | 48 |
| February | 22 | 54 | 18 | 8 | 78 | 62 |
| March | 26 | 14 | 2 | 15 | 47 | 59 |
| April | 21 | 23 | 6 | 7 | 82 | 30 |
| May | 12 | 24 | 0 | 0 | 41 | 111 |
| June | 10 | 0 | 13 | 0 | 20 | 0 |
| July | 1 | 0 | 10 | 21 | 71 | 18 |
| August | 4 | 0 | 8 | 0 | 16 | 65 |
| September | 4 | 0 | 6 | 0 | 63 | 0 |
| October | 18 | 0 | 9 | 0 | 24 | 4 |
| November | 24 | 0 | 0 | 0 | 46 | 0 |
| December | 3 | 10 | 0 | 3 | 47 | 50 |
| Total | 196 | 160 | 79 | 56 | 586 | 447 |
| Standard dev. | 15.70 |  | 6.3 |  | 29.0 |  |
| % Consistency | 81.60% (160/196) | | 70.90% (56/79) | | 76.30% (447/586) | |

Overall % consistency =77% (663/861) – [target; ≥95%]

Overall % discrepancy=23% (198/861) – [target; <5%]

315 Key: **S1**: Number of samples sent from St Benedict’s Ndanda Hospital (SBNH) to National Public 316 Health Laboratory (NPHL) by using SBNH sentinel site book, **S2**: Number of samples sent from 317 SBNH to NPHL by using NPHL sample receiving Laboratory Information System (LIS), **S3**: 318 Number of samples sent from Dodoma Regional Referral Hospital (DRRH) to NPHL by using 319 DRRH sentinel site book, **S4**: Number of samples sent from DRRH to NPHL by using NPHL 320 sample receiving LIS, **S5**: Number of samples sent from Mwananyamala Regional Referral

Hospital (MRRH) to NPHL by using MRRH sentinel site book, **S6**: Number of samples sent from MRRH to NPHL by using NPHL sample receiving LIS.

# Flexibility

Through document review it was revealed that, the system is flexible after standard case definitions has been revised in 2011 to comply with WHO’s standards and proves to work through the system[10]. Also, the system through influenza testing algorithm, allows expansion to include a wider range of other emerging influenza viruses for patients with other respiratory illness other than emerging and re-emerging Influenza A and B viruses. The system was also able to detect and accommodate the severe acute respiratory syndrome coronavirus 2 (SARS-COV-2) in 2019. The mean score for

flexibility was 5.

# Representativeness

Sentinel sites were chosen representatively (not randomly) based on, population density, climatic and socio diversity in the country so as to facilitate estimation of disease burden in the whole population. Data are captured from 14 sentinel sites found in 11 regions of Tanzania (see Fig 1). Furthermore, the distribution of people under surveillance involved all age groups, where by 55.1% (953/1731) of all people under surveillance were children aged under five years (see Table 1); whereas, the lowest coverage [(3.1%)53/1731] was seen in elders above 65 years. This shows that the system is well representative. The mean score for representativeness was 4.

# Timeliness

Turnaround time (TAT) is the time required from receipt of samples by the NPHL, processing, testing and until the dispatch of results. The NISSS protocol in Tanzania, describes this time is 7 days. Among all samples requested for the real-time reverse transcription polymerase chain reaction (RT -PCR) influenza test, only 36.6%

(633/1731) met the required TAT (Target is ≥90%). The mean score for timeliness was

3.

# Acceptability

Despite the fact that, the interviewed stakeholders accepted the operation of the system by 100% (12/12), the results from other attributes compromises their acceptance. This is because timeliness of results, was observed to only 36.6% of the results (target ≥90%), Sixty percent (60%) of selected forms (251/415) were completely filled (target ≥90%). And when consistency was assessed, the data found in sentinel sites did not match with those found at NPHL in the same period, also; only 21.4% (600/2803) of samples were collected among all admitted SARI cases. According to these results obtained, it shows that the acceptability of personnel to participate fully in the NISSS is poor. The mean score for acceptability of the NISSS was 4.

# Sensitivity

The NISSS was sensitive since it has been able to detect cases of influenza and was capable of confirming an outbreak of influenza (A-H1N1 in 2009). Furthermore, the

NISSS was also able to detect other respiratory viruses including, SARS-COV2 in 2019.

The mean score for the sensitivity of the NISSS was 5.

# Predictive value positive (PVP)

Predictive value positive (PVP) of the influenza surveillance system is the proportion of reported cases of influenza that actually have influenza (i.e., the disease under surveillance). The number of reported cases of influenza who actually had influenza in 2019, was 373. And therefore; PVP was 21.5 % (373/1731) and the target ≥90%. This data shows that the PVP of the system is very low (as the system was able to identify only 21.5% true cases among all cases reported by the system). The mean score for PVP of the NISSS was 2.

# Stability

The system utilizes employed staff at all levels. The system is financially stable, since it is funded by WHO, and United States government through Centres for Disease Control and Prevention, and is also supported by Ministry of Health of Tanzania. The mean score for stability of the NISSS was 4.

**Table 4: Findings from the evaluation of the National Influenza Sentinel Surveillance System in Tanzania,2019.**

| **System attribute and definition** | **Method of evaluation** | **Tools used** | **Indicators** | **Score (%)** | **Mean**  **score (%)** | **Tar get**  **scor e (%)** |
| --- | --- | --- | --- | --- | --- | --- |
| **Utility/Usefulness**    How does the system provide information that is useful for public health authorities and communities | - Document review - Observation - Interview of stakeholders - Questionnaires | - Periodic reports and bulletin - Case definition   (SARI, ILI)   - Interview guide  Case- registers |  Importance of  the surveillance system | 5 (80) | 5 (92) | 5  (≥8  0) |
|  |  |  |  Provided  useful data | 5 (90) |  |  |
|  |  |  |  Proportion of surveillance staff that reported that they regularly receive the  Monthly Influenza bulletin | 5 (100) |  |  |
|  |  |  |  Annual  Influenza report | 5 (100) |  |  |
|  |  |  |  Identification and sharing of circulating seasonal influenza strains | 5 (80) |  |  |
|  |  |  |  Outbreaks  detected over the pre- | 5 (100) |  |  |

|  |  |  |  |  |  | established threshold |  |  |  |
| --- | --- | --- | --- | --- | --- | --- | --- | --- | --- |
| **Simplicity**    Refers to both structure and ease of operation of a public health surveillance system |      | Document review  Observation Interview of stakeholders |        | Periodic reports  Case definition  (SARI, ILI)  Interview guide  Case- registers |  | Proportion of samples collected from admitted  SARI cases [**N**: Number of samples collected from admitted  SARI cases; D: Total number of cases that met SARI case definition] | 2 (21) | 3 (57) | 5  (≥8  0) |
|  |  |  |  |  |  | Data collection and shipment | 3 (50) |  |  |
|  |  |  |  |  |  | Perception of stakeholders on simplicity of the system | 5 (100) |  |  |
| **Flexibility**    The ability of a surveillance system to change information needs or operating conditions with little additional time, personnel, or allocated funds |      | Document review  KI-interview  Observation |    | Case-investigation flow chart  Interview guide |  | Changes in standard case definition | 5 (100) | 5  (100) | 5  (≥8  0) |
|  |  |  |  |  |  | New illnesses revealed by the system | 5 (100) |  |  |
|  |  |  |  |  |  | Inclusion of other pathogens surveyed with influenza surveillance system | 5 (100) |  |  |
| **Representativeness** |    | Document review  Observation |    | Policy /guidelines  System reports |  | Total number of cases  captured | 3 (50) | 4 (64) | 5  (≥8  0) |

| Describes the occurrence of a health-related event over time and its distribution in the population by place and person |  KI-interview |  | Surveillance system protocol |  | Number of sentinel sites involved | 4 (60) |  |  |
| --- | --- | --- | --- | --- | --- | --- | --- | --- |
|  |  |  |  |  | Geographical coverage | 3 (44) |  |  |
|  |  |  |  |  | Inclusion of  all age groups | 5 (100) |  |  |
| **Timeliness**    Reflects the speed between steps in a public health surveillance system | - Document review - Interview of stakeholders |      | Laboratory request forms  Laboratory report forms  Specimen registers |  | Proportion of timely reporting of results **[N:** Number of  results reported within required TAT (within 1 week); **D:** Total number of results reported at a time period]. | 3 (55) | 3 (55) | 5  (≥9  0) |
| **Data quality**    The completeness and validity of the data recorded in the public health surveillance system | |  |  |  |  |  |  |  |
| **i. Completeness** |  Document review |    | Case based forms  (CBFs)  Periodic reports |  | Percentage of blank forms **[N:** Number of incomplete filled forms; **D:** Total Number of evaluated forms] | 3 (40) | 3 (40) | 1  (<1  0) |
|  |  |  |  |  | Percentage of complete filled CBFs/ reports **[N:** Number of | 3 (60) | 3 (60) | 5  (≥9  0) |

|  |  |  |  |  |  |  | complete filled forms;  **D:** Total number of  evaluated  forms Case based forms and/or periodic reports]. |  |  |  |
| --- | --- | --- | --- | --- | --- | --- | --- | --- | --- | --- |
| **ii.** | **Data accuracy** |      | Document review  Observation  KI-interview |      | External Quality Assessment (EQA records)  Internal Quality control (IQC) records  Nonconformance  records |  | % EQA  performance **[N:** Number of EQA panel with performance ≥90%; **D:** Total number of EQA panels evaluated]. | 5 (100) | 5  (100) | 5  (≥9  0) |
|  |  |  |  |  |  |  | % Of IQC passed within a selected time period **[N:** Total number of  IQC passed; **D:** Total number of IQC  evaluated]. | 5 (100) | 5  (100) | 5  (100  ) |
| **iii.** | **Data consistency** |      | Document review  Observation  KI-interview |      | Sample register books (from sentinel sites and  National Influenza  Center (NPHL)) Sample transportation log Interview guide |  | %  Consistency between sample sent from selected sentinel sites to NPHL to | 4 (77) | 4 (77) | 5  (≥9  5) |

|  |  |  |  |  |  | those received at NPHL  [Proportion of the difference in number of samples sent from sentinel sites to NPHL to the number of the same samples received at  NPHL] |  |  |  |
| --- | --- | --- | --- | --- | --- | --- | --- | --- | --- |
| **Acceptability**    The willingness of persons and organizations to participate in the surveillance system |      | Document review  Interview of stakeholders  Observation |    | Interview guide Documents and evaluation for evaluation of other attributes |  | Performance observation from other attributes | 3 (50) | 4 (75) | 5  (≥9  5) |
|  |  |  |  |  |  | Perception of stakeholders on accepting the system [**N:** Number  of stakeholders accepting the system; **D:** Total number of stakeholders evaluated via interview]. | 5 (100) |  |  |
| **Sensitivity**    The ability of the surveillance system to detect an outbreak |  | Document review |      | SARI/ILI case definitions  Guidelines Periodic reports and bulletin |  | Proportion of the number of outbreaks identified by the system [**N:** Number of outbreaks detected by the system; **D:** Total number of | 5 (100) | 5  (100) | 5  (≥9  0) |
|  |  |  |  |  |  | outbreaks occurred period]. |  |  |  |
| **Predictive value Positive**  **(PVP)**    proportion of reported cases of influenza that have influenza (i.e., the disease under surveillance). |  | Document review |    | Testing reports  Results registers |    | Proportion of cases correctly picked by the surveillance system (TP); Number of healthy individuals picked as cases by the system (FP). [**N:** Number  of cases truly identified by the system (TP); **D:** Total number of cases identified by the surveillance system  (TP+FP)]. | 2 (21) | 2 (21) | 5  (≥9  0) |
| **Stability**    The reliability and availability of the public health surveillance system |  | Document review |    | System Guidelines Interview guide/questionnair es |    | Number of funding sources of the system Presence of internal and/or external funding mechanism | 4 (80) | 4 (80) | 5  (≥8  0) |

**Key:** Surveillance performance description: a scale from 1 to 5 was used to provide a score for each indicator with a percentage value as follows:

[0–20% [score 1 (very poor performance); [20–40% [score 2 (poor performance); [40–60% [score 3 (moderate performance); [60–80% [score 4 (good performance); [80–100% [score 5 (very good performance). These indicators were scored from 1 to 5 but these scores were based on the consensus opinions of surveillance experts: virologist public health specialist and epidemiologists Who are also working at the National Influenza Sentinel Surveillance System. (N: Numerator, D: Denominator, KI-Key informant interview).

**Risk factors associated with influenza positivity among people enrolled in the national influenza surveillance system in Tanzania.**

Several factors including age, sex, case definition type (ILI and SARI), and turnaround

time (TAT) were assessed for their association with the influenza disease (see Table 5).

Logistic regression analysis was done and showed that sex had no association with

influenza disease [aOR=0.87(95% CI: 0.69-1.10); p=0.252]. We also found that

Children aged less than 5 years old had 56% less likelihood of having influenza as

compared to those aged 65 years and above [aOR=0.44(95% CI: 0.24-0.80); p=0.007].

Case definition type (ILI or SARI) was also associated with influenza whereby, clients

who had ILI symptoms were 1.2 times more likely to be detected for influenza disease

than those presented with SARI [aOR=1.36 (95%CI: 1.03-1.79); p=0.031]. In addition,

laboratory results with TAT more than 7 days were 1.5 times more likely to be detected 406 for influenza as compared to those processed and dispatched within 7 407 days[aOR=1.52(95% CI:1.18-1.97); p=0.001] (see Table 5).

| **Variable** | **Influenza results** | | | **Bivariate** | | | **Multivariate** | |
| --- | --- | --- | --- | --- | --- | --- | --- | --- |
|  | Negative | Positive | COR (95% CI) | | P value | AOR (95% CI) | | P value |
| Sex  Female | 615 (76.5) | 189 (23.5) | 1 (Ref) | |  | 1 (Ref) | |  |
| Male | 727 (80.3) | 178 (19.7) | 0.80 (0.63-1.00) | | 0.054 | 0.87 (0.69-1.10) | | 0.252 |
| Age (in years)  0-4 | 779 (82.8) | 162 (17.2) | 0.40 (0.22-0.73) | | 0.003 | 0.44 (0.24-0.80) | | **0.007** |
| 5-14 | 92 (71.9) | 36 (28.10 | 0.76 (0.38-1.51) | | 0.435 | 0.71 (0.35-1.42) | | 0.329 |
| 15-24 | 114 (71.7) | 45 (28.3) | 0.77 (0.39-1.49) | | 0.436 | 0.70 (0.36-1.38) | | 0.309 |
| 25-34 | 137 (72.9) | 51 (27.1) | 0.72 (0.38-1.39) | | 0.332 | 0.65 (0.33-1.26) | | 0.201 |
| 35-44 | 89 (78.8) | 24 (21.2) | 0.52 (0.25-1.08) | | 0.081 | 0.49 (0.23-1.02) | | 0.055 |
| 45-54 | 45 (75.0) | 15 (25.0) | 0.65 (0.29-1.46) | | 0.297 | 0.61 (0.27-1.38) | | 0.234 |
| 55-64 | 51 (76.1) | 16 (23.9) | 0.61 (0.27-1.36) | | 0.225 | 0.57 (0.25-1.28) | | 0.175 |
| ≥65 | 35 (66.0) | 18 (34.0) | 1 (Ref) | |  | 1 (Ref) | |  |
| Case definition  ILI | 752 (74.9) | 252 (25.1) | 1.72 (1.34-2.20) | | <0.001 | 1.36 (1.03-1.79) | | **0.031** |
| SARI | 590 (83.7) | 115 (16.3) | 1 (Ref) | |  | 1 (Ref) | |  |
| Turnaround time  >7 | 816 (75.6) | 264 (24.4) | 1.65 (1.28-2.13) | | <0.001 | 1.52 (1.18-1.97) | | **0.001** |
| ≤ 7 | 526 (83.6) | 103 (16.4) | 1 (Ref) | |  | 1 (Ref) | |  |

**Table 5. Logistic regression analysis on the factors associated with influenza** 421	**positivity among people enrolled in National influenza surveillance system in**	**Tanzania from January to December, 2019 (n=1709).**

# Discussion

This study assessed the National Influenza Sentinel Surveillance System in Tanzania, to provide comprehensive data on strengths, weaknesses and provide suitable recommendations for improvement.

The observed distribution of the influenza disease by sex, influenza types and case definition type, from the NISSS in Tanzania, has not shown any significant difference from the study done by Mmbaga et al, in the year, 2012[5].

This evaluation report has clearly shown that the NISSS in Tanzania has an overall moderately performance (with mean score of 3 to 4). Although the PVP was low [21.5% (target ≥90%)], this is relatively higher compared to PVP of 7.9% obtained from the previous evaluation report, on 30 months of evaluation after establishment of the NISSS in Tanzania[5]. The observed inconsistency of data from the sentinel sites to National Influenza Laboratory (that is 23% data discrepancy compared with the target of <5%) is most likely due to errors during documentation at the sentinel sites. The observed higher TAT [only 37% (target ≥90%)], is likely due to prolonged registration time at NPHL, which may be due to a shortage of human resources, who could hasten

registration process of case-based forms in LIS before testing.

From this evaluation, we report that; the prevalence of influenza A (with circulating influenza A subtype; H3N2), is relatively higher than that of influenza B regardless of the case definition type used, sex or age category of the individuals under surveillance. The same results have been shown by several literatures in evaluation of influenza surveillance in different parts of the world[5–7, 11]. Furthermore, clients who had ILI symptoms had 1.2 times higher odds of being detected for influenza disease than those presented with SARI [aOR=1.36 (95%CI: 1.03-1.79); p=0.031]. This could be explained by uneven distribution of ILI and SARI cases among patients under influenza surveillance as shown in the previous evaluation report[5].

The timeliness of the NISSS in Tanzania, may have been affected by the geographical distribution of sentinel sites and the simplicity of data collection and reporting methods.

An electronic information management system (from the sentinel sites level to the NPHL or NIC), was one of the most important recommendations proposed by

surveillance staff to improve data completeness, quality monitoring and timeliness. This would facilitate the real-time sharing of data at all levels involved in the NISSS[12]. The evaluation of syndromic influenza sentinel surveillance system in Madagascar between 2009 and 2014 showed that by using mobile phones and texting for the transmission of daily aggregated data, the simplicity of the system was strengthened, improving the completeness, quality and timeliness of the data as well as acceptability of the system to the sentinel site staff[13].

This is in line with the recommendation that a revised data management approach, which offers the advantage of automation and user-friendly interface between the sentinel sites and the NIC, would ultimately improve the acceptance and utility of the surveillance system.

Although system representativeness was moderate (64%) due to involvement of all age category in the surveillance, number of sentinel sites are still inadequate (only 14 sentinel sites available) to cover the entire population of Tanzania and thus, increasing more sentinel sites could have increased the coverage and the level of system representativeness. The National Influenza Technical Working Group (NITWG) should plan for reviewing the current case definition to improve the PVP, ensure that the NISSS enrols the true cases of influenza, to minimize misuse of the surveillance resources.

In this study we did not find any significant association of sex with influenza results. This is different from the study done by Guerrisi C et al, 2019 that showed that; females are the most affected group [aOR=1.29 (95% CI: 1.20-1.40)], but this association could be also affected by the disproportionate distribution among female and male individuals under surveillance system, as for this, 61% of the total study participants were the female group[14].

Although age was associated with influenza positivity, children aged less than 5 years old was the only age group that showed significant association and their distribution was higher compare to the rest of other age groups. In addition, laboratory results with TAT more than 7 days had 1.5 times higher odds of being detected for influenza as compared to those processed and dispatched within 7 days[aOR=1.52(95% CI:1.18-

1.97); p=0.001].This might be due to more time required for further characterization of the influenza subtypes and insufficient human resource at NPHL-influenza unit.

From a virologic perspective, the surveillance of influenza in 2019 permitted the characterization of circulating influenza viruses, and ensured strong links with the WHO Collaborating Centers on influenza, by participating in vaccine strain selection and shipping specimens twice a year, as recommended by WHO.

# Conclusion

The system performed overall satisfactorily in compliance with its objectives and generates accurate data with an average performance of 100%; which entails a realistic estimation of the burden of influenza disease in the country and which will help the country to plan for prioritizing responding to the disease outbreaks and even plan for the vaccine development in the future. The complexity of the system contributed to its data inconsistency. Better use of the available data could be made to inform and promote preventive measures, especially among the most vulnerable population (i.e., children aged <5years). Increasing sentinel sites could be made to maximize coverage and level of system representativeness.

# Limitations of the study

The sentinel sites used manual registers for registration of surveillance individuals while NPHL or NIC uses electronic system (Disa Lab). Since these are two different systems, may have contributed to the high data discrepancy between NIC and sentinel sites. In addition, some of influenza A virus detected [80% (230/284)] were not characterized into subtypes.

**Supporting documents:**

***S1 Fig. Data flow of the National Influenza Sentinel Surveillance System in Tanzania***

***S1 File. Data collection tool for the evaluation of National Influenza Surveillance System in Tanzania***

***S2 File. Updated guidelines for evaluating public health surveillance systems* Acknowledgements**

Sincere appreciation to NPHL, Tanzania field epidemiology and laboratory training program (TFELTP), Muhimbili university of health and allied science (MUHAS), MoH- epidemiology section, for their great support in this fieldwork. Special thanks are also granted to WHO-emergency preparedness and response, Dar es salaam, Tanzania, as well as SBNH, DRRH and MRRH, for allowing me to conduct this fieldwork in their

sites.

# Authors’ contributions

All authors have read and approved the manuscript and take responsibility for the integrity of the data and the accuracy of the data analysis. *Study concept and design*: Vulstan Shedura, Geofrey Mchau, Doreen Kamori *interviewing of stakeholders and software use:* Vulstan Shedura, Salum Nyanga, Geofrey Mchau; *Data cleaning, analysis and interpretation*: Vulstan Shedura, Doreen Kamori, Ally Hussein; *Drafting of manuscript*: Vulstan Shedura, Geofrey Mchau, Doreen Kamori

**Funding**

This work had no official funding.

# Competing interests

All authors declare that they have no commercial or other associations that may pose a conflict of interest.

# References

[1] WHO Global Influenza Programme Surveillance and Epidemiology team., “Protocol for National Influenza Sentinel,” *Isbn*, p. 2, 2015.
[2] World Health Organization, *National Protocol for Influenza Sentinel Surveillance System*, First edit. Geneva: World Health Organisation, Geneva, 2015.
[3] P. Babakazo *et al.*, “Evaluation of the influenza sentinel surveillance system in the Democratic Republic of Congo, 2012-2015,” *BMC Public Health*, vol. 19, no. 1, pp. 1–12, Dec. 2019, doi: 10.1186/S12889-019-8008-2/TABLES/5.
[4] WHO, “WHO | Influenza (Seasonal),” *Bulletin of the World Health Organization*, 2013. .
[5] V. M. Mmbaga *et al.*, “Results From the First 30 Months of National Sentinel Surveillance for Influenza in Tanzania, 2008–2010,” *J. Infect. Dis.*, vol. 206, no. suppl_1, pp. S80–S86, Dec. 2012, doi: 10.1093/infdis/jis540.
[6] E. Budgell *et al.*, “Evaluation of two influenza surveillance systems in South Africa,” *PLoS One*, vol. 10, no. 3, Mar. 2015, doi:

10.1371/journal.pone.0120226.

[7] F. S. Nuvey *et al.*, “Evaluation of the sentinel surveillance system for influenza-like illnesses in the Greater Accra region, Ghana, 2018,” *PLoS One*, vol. 14, no. 3, p. e0213627, Mar. 2019, doi:

10.1371/JOURNAL.PONE.0213627.

[8] R. Yazidi *et al.*, “Evaluation of the influenza-like illness surveillance system in Tunisia, 2012-2015,” *BMC Public Health*, vol. 19, no. 1, pp. 1–9, Jun. 2019, doi:

10.1186/S12889-019-7035-3/FIGURES/2.

[9] National Bureau of Statistics Ministry of Finance, “The United Republic of Tanzania 2012 Population and housing census- Population Distribution by Administrative Areas,” 2013.
[10] W. H. Organization, *PROTOCOL FOR NATIONAL INFLUENZA SENTINEL*

*SURVEILLANCE Integrated Disease Surveillance Programme Health Security and Emergencies Cluster*. .

[11] P. O. Adogu, C. I. Achebe, and C. F. Ubajaka, “Epidemiologic study of influenza infection in a developing country-experience in a tertiary care center in South East Nigeria,” *J. Med. Med. Sci.*, vol. 5, no. 3, pp. 61–70, 2014, doi:

10.14303/jmms.2014.040.

[12] D. M. Sosin, “Draft framework for evaluating syndromic surveillance systems,” *J. Urban Health*, vol. 80, no. 2 Suppl 1, 2003, doi:

10.1007/PL00022309.

[13] A. Rakotoarisoa *et al.*, “Lessons from the field Evaluation of the influenza sentinel surveillance system in Madagascar,” *Bull World Heal. Organ*, vol. 95, pp. 375–381, 2017, doi: 10.2471/BLT.16.171280.
[14] C. Guerrisi *et al.*, “Factors associated with influenza-like-illness: A crowdsourced cohort study from 2012/13 to 2017/18,” *BMC Public Health*, vol.

19, no. 1, pp. 1–9, Jul. 2019, doi: 10.1186/S12889-019-7174-6/TABLES/3.

Fig 1. Sentinel sites of the National Influenza Sentinel Surveillance System in Tanzania, 160 2019 (N=16).

Fig 2. Distribution of influenza types in Tanzania according to case definition used 200 among confirmed cases of influenza from January to December 2019 (N=373).

Fig 3. Weekly distribution of influenza cases in Tanzania from January to December 2019 221 (N=373).

Fig 4. The proportion of incompleteness of selected parameters on influenza casebased 260 forms used by National Influenza Sentinel Surveillance System in Tanzania,

S1 Fig. Data flow of the National Influenza Sentinel Surveillance

System in Tanzania

[Click here to access/download](https://www.editorialmanager.com/pone/download.aspx?id=31286494&guid=19237cee-4fe4-477c-8efa-75ae39b37205&scheme=1)

[Supporting Information S1_Figure.tif](https://www.editorialmanager.com/pone/download.aspx?id=31286494&guid=19237cee-4fe4-477c-8efa-75ae39b37205&scheme=1)

S1 File. Data collection tool for the evaluation of National Influenza

Surveillance System in Tanzania

[Click here to access/download](https://www.editorialmanager.com/pone/download.aspx?id=31286498&guid=0b14552c-d053-4639-978e-dba2bfb84f6b&scheme=1)

[Supporting Information](https://www.editorialmanager.com/pone/download.aspx?id=31286498&guid=0b14552c-d053-4639-978e-dba2bfb84f6b&scheme=1)

[S1_File.pdf](https://www.editorialmanager.com/pone/download.aspx?id=31286498&guid=0b14552c-d053-4639-978e-dba2bfb84f6b&scheme=1)

S2 File. Updated guidelines for evaluating public health surveillance systems

[Click here to access/download](https://www.editorialmanager.com/pone/download.aspx?id=31286499&guid=a771c7fe-4f83-40da-ade5-666bb92f67cf&scheme=1)

[Supporting Information](https://www.editorialmanager.com/pone/download.aspx?id=31286499&guid=a771c7fe-4f83-40da-ade5-666bb92f67cf&scheme=1)

[S2_File.pdf](https://www.editorialmanager.com/pone/download.aspx?id=31286499&guid=a771c7fe-4f83-40da-ade5-666bb92f67cf&scheme=1)
