# Supplementary material for: Evaluation of the influenza-like illness sentinel surveillance system: A national perspective in Tanzania from January to December 2019
Source: PLoS One. 2023 Mar 20;18(3):e0283043. doi: 10.1371/journal.pone.0283043 (PMC10027206; doi:10.1371/journal.pone.0283043)
Supplement: Attachment — Submitted filename: Rebuttal Letter_Response to Reviewers.docx [file pone.0283043.s008.docx]

**Rebuttal letter**

**Response to reviewers**

We thank the editor and reviewers for evaluating our manuscript. We provide line-by-line responses to comments raised. Please, follow our responses in a green mark. to every comment/question in yellow mark. We copied the review decision from the submission menu of the editorial manager. We used the same letter to write our responses.

PONE-D-22-16315R1

Evaluation of the influenza-like illness sentinel surveillance system: A national perspective in Tanzania from January to December 2019.

PLOS ONE

Dear Editor,

Please find the modified manuscript. We have incorporated all suggestions recommended by the reviewers. Following is the point-wise reply to the reviewers' comments.

With best regards,

Mr. Shedura

**Reply to comments**

Comment:

Dear Dr. Shedura,

Thank you for submitting your manuscript to PLOS ONE. After careful consideration, we feel that it has merit but does not fully meet PLOS ONE’s publication criteria as it currently stands. Therefore, we invite you to submit a revised version of the manuscript that addresses the points raised during the review process.

Please submit your revised manuscript by Jan 27 2023 11:59PM. If you will need more time than this to complete your revisions, please reply to this message or contact the journal office at [plosone@plos.org](mailto:plosone@plos.org). When you're ready to submit your revision, log on to <https://www.editorialmanager.com/pone/> and select the 'Submissions Needing Revision' folder to locate your manuscript file.

Response: Thank you for the comments.

Comment:
Please include the following items when submitting your revised manuscript:

A rebuttal letter that responds to each point raised by the academic editor and reviewer(s). You should upload this letter as a separate file labeled 'Response to Reviewers'.

Response: The rebuttal letter has been uploaded as a separate file labeled 'Response to Reviewers'.

Comment:

A marked-up copy of your manuscript that highlights changes made to the original version. You should upload this as a separate file labeled 'Revised Manuscript with Track Changes'.

Response: The revised marked copy of the manuscript that highlights changes made has been uploaded as a separate file labeled ‘revised Manuscript with track changes.

Comment:

An unmarked version of your revised paper without tracked changes. You should upload this as a separate file labeled 'Manuscript'.

Response: Unmarked manuscript without tracked changes has been uploaded as a separate file labeled ‘Manuscript’.

Comment:

If you would like to make changes to your financial disclosure, please include your updated statement in your cover letter. Guidelines for resubmitting your figure files are available below the reviewer comments at the end of this letter.
Response: We have made no changes in financial disclosure statement.

Comment:
If applicable, we recommend that you deposit your laboratory protocols in [protocols.io](http://protocols.io/) to enhance the reproducibility of your results. Protocols.io assigns your protocol its own identifier (DOI) so that it can be cited independently in the future. For instructions see: <https://journals.plos.org/plosone/s/submission-guidelines#loc-laboratory-protocols>. Additionally, PLOS ONE offers an option for publishing peer-reviewed Lab Protocol articles, which describe protocols hosted on [protocols.io](http://protocols.io/). Read more information on sharing protocols at <https://plos.org/protocols?utm_medium=editorial-email&utm_source=authorletters&utm_campaign=protocols>.

Response: We have deposited the laboratory protocols used in [protocols.io](http://protocols.io/). We used the updated guidelines for evaluation of the public health surveillance systems, available at https://stacks.cdc.gov/view/cdc/13376.

Comment:
We look forward to receiving your revised manuscript.

Kind regards,

Clement Adebajo Meseko, DVM, PhD
Academic Editor
PLOS ONE

 Response: Thank you for the comments.

**Journal Requirements:**

Comment:

Please review your reference list to ensure that it is complete and correct. If you have cited papers that have been retracted, please include the rationale for doing so in the manuscript text, or remove these references and replace them with relevant current references. Any changes to the reference list should be mentioned in the rebuttal letter that accompanies your revised manuscript. If you need to cite a retracted article, indicate the article’s retracted status in the References list and also include a citation and full reference for the retraction notice.

 Response: The references have been reviewed.

**Additional Editor Comments:**

Comment:

The manuscript is an important public health surveillance data for influenza and other respiratory viruses but still in need of clarity and improvement including grammar. Note the comments by both reviewers that subtype H5 is not a seasonal influenza and it is important to clarify if this is a valid detection because of the importance of H5 in global public health and influenza virus reporting to the WHO.
[Note: HTML markup is below. Please do not edit.]

 Response: It is true that H5 subtype is Avian not seasonal influenza, thank you for this observation. Although it was part of the included investigations, we have decided to exclude it so as to align with the objectives of our study. We have stated that the Influenza subtype involved are H1, H3 and H1N1pdm09.

**Reviewers' comments:**

**Reviewer's Responses to Questions**

**Comments to the Author**

Comment:

1. If the authors have adequately addressed your comments raised in a previous round of review and you feel that this manuscript is now acceptable for publication, you may indicate that here to bypass the “Comments to the Author” section, enter your conflict of interest statement in the “Confidential to Editor” section, and submit your "Accept" recommendation.

Reviewer #1: All comments have been addressed

Reviewer #2: (No Response)

Response: Thank you for the comments

Comment:

2. Is the manuscript technically sound, and do the data support the conclusions?

The manuscript must describe a technically sound piece of scientific research with data that supports the conclusions. Experiments must have been conducted rigorously, with appropriate controls, replication, and sample sizes. The conclusions must be drawn appropriately based on the data presented.

Reviewer #1: Yes

Reviewer #2: Yes

Response: Thank you for the comments

Comment:

3. Has the statistical analysis been performed appropriately and rigorously?

Reviewer #1: Yes

Reviewer #2: No

Response: Thank you for the comments

Comment:

4. Have the authors made all data underlying the findings in their manuscript fully available?

The [PLOS Data policy](http://www.plosone.org/static/policies.action#sharing) requires authors to make all data underlying the findings described in their manuscript fully available without restriction, with rare exception (please refer to the Data Availability Statement in the manuscript PDF file). The data should be provided as part of the manuscript or its supporting information, or deposited to a public repository. For example, in addition to summary statistics, the data points behind means, medians and variance measures should be available. If there are restrictions on publicly sharing data—e.g. participant privacy or use of data from a third party—those must be specified.

Reviewer #1: Yes

Reviewer #2: Yes

Response: Thank you for the comments

Comment:

5. Is the manuscript presented in an intelligible fashion and written in standard English?
PLOS ONE does not copyedit accepted manuscripts, so the language in submitted articles must be clear, correct, and unambiguous. Any typographical or grammatical errors should be corrected at revision, so please note any specific errors here.

Reviewer #1: Yes

Reviewer #2: No

Response: Thank you for the comments. We have corrected all typographical and grammatical errors in the revised manuscript.

Comment:

6. Review Comments to the Author
Please use the space provided to explain your answers to the questions above. You may also include additional comments for the author, including concerns about dual publication, research ethics, or publication ethics. (Please upload your review as an attachment if it exceeds 20,000 characters)

Reviewer #1: Line 132 All specimens positive for influenza A virus were subtyped for seasonal H1(A[H1], H3(A[H3]), H5(A[H5]) and A(H1N1) pdm09
The () are not correctly distributed and H5 is not a seasonal subtype but avian, it should read :All specimens positive for influenza A virus were subtyped for H1, H3, H5 and H1N1pdm09
Yet is this really so? H5 subtyping is carried out in sentinel seasonal influenza surveillance?

Response: It is true that H5 subtype is avian not seasonal influenza, thank you for this observation. Although it was part of the included investigations, we have decided to exclude it so as to align with the objective of our study. We have stated that the Influenza subtype involves H1, H3, and H1N1pdm09.

Comment:

Reviewer #2: English needs to be reviewed, especially in modified text (in red in manuscript with corrections). New or rewritten parts need to be checked thoroughly. The sentences are sometimes clumsy.
The description of the evaluation criteria is very useful and enhances greatly the manuscript. The authors should however now work on simplifying the manuscript. Perhaps that the criteria and the results could be extensively presented in a table (i.e. table 3 with additional information if needed), but maybe less extensively in the text.

Response: Thank you for the comments. We have corrected all typographical and grammatical errors in the revised manuscript. We have simplified the Manuscript in the revised version.

Comment:
“clients” is still used several times in the manuscript.
Laboratory procedures: H5 is not considered as a seasonal human influenza viruses.

Response: We have removed the word “clients” throughout the manuscript and replaced it with the word “patents”. We have removed the avian influenza subtype (H5) in the revised manuscript to make it clear pertaining to seasonal influenza sentinel surveillance.

Comment:
Predictive Positive Value: it is still not clear how the authors were able to calculate this. This would require to have the number of influenza positive and negative samples among cases (matching the case definitions) and non-cases. The surveillance system aims at enrolling only cases. The authors have thus access only to True Positive and False Negative (what the authors wrongly call False Positive).

Response: Thank you for the comments. It is true that the surveillance system aims at enrolling only cases, but in influenza surveillance, there are ***suspected cases*** and ***confirmed cases***. All cases enrolled in NISSS are ***suspected cases*** of influenza that meet either the ILI or SARI case definition. Samples are drawn from all suspected cases for laboratory confirmation and those who will be detected positive are the confirmed cases (true positives) and those who will detect negative after confirmation are the “false positives”. According to the updated guidelines for evaluating public health surveillance systems available at <https://stacks.cdc.gov/view/cdc/13376>.(pages; 18-20), defines the Predictive value positive (PVP) of the surveillance system as the proportion of reported cases that actually have the health-related event under surveillance. For this context, the health-related event under surveillance is Influenza disease. Furthermore; from the cited guideline, the calculation of the PVP is done by using a formula: True positive/ (True positive + False negative). The “True positive” of the surveillance system are those patients who met the standard case definition of either SARI or ILI, depending on their clinical presentations, and therefore enrolled in to the surveillance system, and their PCR results showed positive results of the influenza Virus A or Influenza Virus B. So, in this study such patients were 373 in number. However; the other category of “False positive” of the surveillance system are those patients who met the standard case definitions (SARI/ILI), enrolled in to the surveillance system but their PCR results showed no detection of Influenza virus. For this study such patients were 1348 in number. Therefore; according to the PVP formular; True positive/ (True positive + False negative); PVP=373/ (373+1348) =21.7%. In summary; we calculated the PVP of the Influenza surveillance system and not PVP of the influenza test.

Comment:
The manuscript is really focusing on a major aspect of surveillance: its evaluation. There are not many papers providing such an evaluation, so the manuscript is really important for the scientific community involved in public health surveillance of influenza and other respiratory viruses. It is so important in fact, that it is worth for the authors to take a bit more time to make their manuscript as clear and easy to follow as possible. As such, the content is ok, but the form can be improved in order to avoid too long sentences and to correct the grammar. The clarity of the message will gain from a more synthetic presentation.

Response: Thank you for the comments.

Comment:

7. PLOS authors have the option to publish the peer review history of their article ([what does this mean?](https://journals.plos.org/plosone/s/editorial-and-peer-review-process#loc-peer-review-history)). If published, this will include your full peer review and any attached files.
If you choose “no”, your identity will remain anonymous but your review may still be made public.
**Do you want your identity to be public for this peer review?** For information about this choice, including consent withdrawal, please see our [Privacy Policy](https://www.plos.org/privacy-policy).

Reviewer #1: No

Reviewer #2: No

Response: Thank you for the comments.

Comment:

[NOTE: If reviewer comments were submitted as an attachment file, they will be attached to this email and accessible via the submission site. Please log into your account, locate the manuscript record, and check for the action link "View Attachments". If this link does not appear, there are no attachment files.]
While revising your submission, please upload your figure files to the Preflight Analysis and Conversion Engine (PACE) digital diagnostic tool, <https://pacev2.apexcovantage.com/>. PACE helps ensure that figures meet PLOS requirements. To use PACE, you must first register as a user. Registration is free. Then, login and navigate to the UPLOAD tab, where you will find detailed instructions on how to use the tool. If you encounter any issues or have any questions when using PACE, please email PLOS at [figures@plos.org](mailto:figures@plos.org). Please note that Supporting Information files do not need this step.

Response: Thank you.
